# Density, Climate, and Stochasticity Shape Four Centuries of Population Dynamics for Two Long‐Lived Tree Species

**DOI:** 10.1002/ece3.70664

**Published:** 2024-12-15

**Authors:** Ellen Waddle, Mark R. Lesser, Christopher Steenbock, Daniel F. Doak

**Affiliations:** ^1^ Department of Ecology and Evolutionary Biology University of Colorado Boulder Boulder Colorado USA; ^2^ Center for Earth and Environmental Science SUNY Plattsburgh Plattsburgh New York USA; ^3^ Department of Environmental Studies University of Colorado Boulder Boulder Colorado USA

**Keywords:** climate, colonizing population, *Pinus flexilis*, *Pinus ponderosa*, population dynamics

## Abstract

The dynamics of colonizing populations may be strongly influenced by both extrinsic (e.g., climate and competition) and intrinsic (e.g., density) forces as well as demographic and environmental stochasticity. Understanding the impacts of these effects is crucial for predicting range expansions, trailing edge dynamics, and the viability of rare species, but the general importance of each of these forces remains unclear. Here, we assemble establishment time and spatial locations of most individuals that have reached maturity in six isolated, establishing populations of two pine species. These data allow us to quantify the relative importance of multiple factors in controlling growth of these populations. We found that climate, density, site, and demographic stochasticity were of varying importance both within and across species, but that no driver appeared to dominate dynamics across all populations and time periods. Indeed, exclusion of any one of these effects greatly reduced predictive power of our population growth models. Given the similarity in the abiotic characteristics of these sites, the varying importance of these classes of effects was surprising but speaks to the need to consider multiple effects when predicting the dynamics of small and colonizing populations.

## Graphical abstract

Using four centuries of data on the establishment of long‐lived pines, we compare the explanatory power of climate, density dependence, stochasticity, and spatial dynamics on the growth of colonizing populations. We find that despite the shared climate and proximity to one another, each of the six populations shared few commonalities, and each driver varied in its importance across all populations and time periods. This speaks to the need to explicitly consider density and stochasticity effects, as well as climate, when constructing models to explain dynamics of small and colonizing populations.

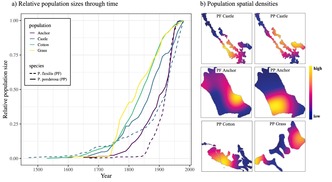

## 1 Introduction

Dating to the controversy between Andrewartha and Birch and Nicholson (Andrewartha and Birch 1954; Cold Spring Harbor Symposium 22 1957), ecologists have debated the roles of extrinsic versus intrinsic forces in shaping population dynamics, a question that continues to this day (Adler et al. 2018; Thibaut and Connolly 2020). The classic posing of this issue involved the roles of density dependence, as an intrinsic force, versus climate variation, as an extrinsic force, in governing changes in population numbers. More recent treatments also consider the roles of demographic and genetic stochasticity as forces that can drive changing numbers, including the differential effects of stochasticity at low versus high population sizes (Lande 1998a, 1998b; Palstra and Ruzzante 2008). In addition, interspecific interactions are often also regarded as extrinsic drivers (Kaplan and Denno 2007), along with climate and other abiotic effects. In this formulation, three separate classes of effects that can drive dynamics are distinguished: (1) extrinsic drivers that include climatic and biotic fluctuations that occur without feedback from the focal population density (i.e., environmental stochasticity); (2) variation that occurs as an inevitable consequence of low numbers (i.e., demographic stochasticity); and (3) variation due to predictable effects of density on vital rates that result in changing, density‐dependent growth rates. Obviously, there is also potential for multiple, complex interactions between these effects (Miller 1995; Song and Corlett 2021).

Understanding the importance of different drivers of dynamics for small populations is of interest for several reasons. First is the simple need to understand what factors are most critical in driving natural population numbers. Second is the desire to predict the dynamics of species of concern for either conservation reasons or resource extraction. A particular question here is how to predict future trends from often limited data on past numbers. A final and related issue concerns the forces that predict growth, stagnation, or extinction of colonizing populations, which are, of necessity, small when they are founded. The dynamics of colonizing populations has long been of interest in invasion ecology but is now also central to the prediction of shifting ranges of native species that must colonize new terrain to keep pace with climate change (Loarie et al. 2009; Hockey et al. 2011). In general, if intrinsic forces predominantly shape population growth rates, we expect population dynamics to be more predictable, since similar populations should follow similar trajectories that will be largely unaffected by outside forcings. Conversely, strong effects of extrinsic drivers or demographic stochasticity are expected to make population numbers more difficult to accurately forecast.

Multiple studies have attempted to either theoretically address the contributions of these forces to population dynamics or to analyze their effects in real or model systems. A major theme in this literature involves the detection of density dependence, which has often relied on analysis of time‐series data (Pollard, Lakhani, and Rothery 1987; Turchin 1990), although purely theoretical analyses have also been of considerable importance (Goodman 1984; Lande 1993, 1998c). The role of density dependence has been investigated in model lab systems (Joshi, Wu, and Mueller 1998; Mueller, Gonzalez‐Candelas, and Sweet 1991), and natural populations systems (Moorcroft et al. 1996; Vucetich and Peterson 2004). Another theme, pioneered by Andrewartha and Birch, has been the analysis of the effects of environmental fluctuations on population dynamics (e.g., Dennis and Otten 2000; Melbourne and Hastings 2008). And of course, combined analyses have sought to disentangle the relative effects of density dependence, demographic stochasticity, and abiotic drivers, with varying degrees of support shown for each of these mechanisms (e.g., Bakker et al. 2009; Fromentin et al. 2001).

Despite this long‐standing attention, efforts to judge the comparative strength of these mechanisms in shaping long‐term population dynamics are still sparse (Brook and Bradshaw 2006), in part due to the need for long‐term data on entire populations to most directly compare these effects. In addition, to powerfully test for density dependence, one should ideally have data across a range of population densities—a tall order for most studies of natural populations.

Here, we use data on isolated populations of two long‐lived pine trees to quantify the extrinsic (e.g., climate) versus intrinsic forces (e.g., density dependence) in shaping dynamics of small and still‐growing populations. Our work uses the ages and spatial locations of reproductive individuals from initial colonization to the present for four isolated *Pinus ponderosa* and two isolated *Pinus flexilis* populations in the Bighorn Basin in north central Wyoming. Past work on a subset of these data has shown that the establishment of trees in these populations has been highly variable over time, with dynamics that do not closely follow simple patterns of exponential or logistic growth, but also did not appear to be driven in a simple or consistent way by climate variation (Lesser 2012). The advantage of this system is that high individual longevity and the relative youth of the populations (~400–500 years) mean that the majority of trees that have reached reproductive size are still alive and can be sampled for most of the populations. In addition, dendrochronological methods allow us to precisely age each individual. Substantial post‐establishment mortality is unlikely to have occurred in any of the six populations; evidence of mortality is observable in this system for long periods of time due to the dryness and low fire regimes in the region (Lesser and Jackson 2012, pers. obs.). Thus, the data on each population encompass population establishment and growth—a period of dynamic change in populations and also one that is of key applied importance. Given low adult mortality, our focus is on the combined processes of reproduction, seed establishment, and survival to a size large enough to be seen in dendroecological analyses of the extant populations. The ability to see all adults of a population allows us to directly test the effects of different demographic drivers over long periods. Additionally, being able to test for density dependence and climate effects in separate populations that share similar abiotic settings allows us to examine how general the strength of different drivers is in shaping population dynamics.

We conduct analyses of the spatial and temporal patterns in establishment of each population to address three general questions: (1) Are population growth predictions based on either density‐dependent effects or climate effects alone able to predict dynamics over ~400 years of population change? (2) To what degree has demographic stochasticity shaped dynamics of these new populations, and does consideration of stochasticity improve our predictions of population growth? (3) How well do patterns of effects generalize across populations or even species? In addition to these basic questions, we also conducted spatial analyses to ask if establishment patterns suggested either positive or negative density dependence.

## 2 Methods—Data Collection

### 2.1 Field Data

The four disjunct populations of *Pinus ponderosa* (ponderosa pine) that we studied co‐occur with populations of *Pinus flexilis* (limber pine), and habitats for both species are similar. We surveyed each of the four *P. ponderosa* populations and two of the four co‐occurring *P. flexilis* populations for a total of six populations. We will refer to each population using PF for *P. flexilis* and PP for *P. ponderosa*, followed by the site name as subscript. Both species are slow‐growing, widely distributed, and wind‐pollinated. While these two species vary in elevational range and climate tolerance across their broader geographic range, they overlap here (1450–2050 masl; Figure S11) and are likely both constrained by the extreme aridity of the landscape, which is otherwise dominated by *Juniperus* spp. Limber pine seeds are primarily dispersed by small mammals and Clark's nutcrackers ( *Nucifraga columbiana* ), while ponderosa pines are wind dispersed, although animal caches of ponderosa pine seeds are known to increase probability of establishment (Vander Wall 2008).

We've confirmed the isolation of these populations with both personal observations and with broader occurrence databases and herbarium records. GBIF data shows only 2 and 12 other documented occurrence sites of *P. ponderosa* and *P. flexilis* , respectively, in the Bighorn Basin—an area that spans ~10,000 mile^2^ (GBIF 2024). In addition to searching GBIF, we also searched herbarium records, and no additional records were found. While we cannot rule out the possibility of other small populations in this area, we feel confident that treating these populations as examples of isolated, colonizing populations is appropriate. In continuous populations of *P. flexilis* in northern Colorado, viable pollen exchange occasionally exceeded 500 m; pollen movement on the order of tens to a hundred kilometers likely occurs but is rare in this system (Schuster and Mitton 2000). Seed movement is generally more constrained; most winged *Pinus* seeds fall within tens of meters of their parent, though far greater distances are occasionally observed (Vander Wall 2023). Clark's nutcracker is known to be a long‐distance disperser of seeds, but this species is not common in this area (15 documented occurrences in Bighorn Basin; GBIF 2024), and the distances between our populations are on par with the tail end of dispersal distance estimates via Clark's nutcracker in other systems (32 km; Lorenz et al. 2011). No other *Pinus* individuals were found within 15 km of the outermost trees, and generally far greater isolation than this (Lesser, pers. obs., Figure S9). However, parentage analyses on these *P. ponderosa* populations showed that all of the first individuals originated from long‐distance dispersal events, and 29%–70% of individuals were not assigned to at least one local parent at 50% confidence level (Lesser 2013). Thus, outside gene flow was likely crucial to population initiation and growth.

At all sites, Ponderosa pines were initially censused and cored between 2006 and 2008, and virtually all were re‐GPSed in 2016–2017 to obtain more accurate positions. Limber pines were censused in 2016 and 2017. Each tree was georeferenced with a handheld GPS, and two cores were taken from every tree. After post‐processing, most positions were accurate to +/− 50 cm. Cores were mounted, sanded, and scanned for ring counts using WinDENDRO (Regent Instruments 2008). Cores were cross‐dated using skeleton plotting and correlation analysis, and ages were then adjusted for coring height (see Lesser and Jackson 2012 for more detail). The field data consist of spatial locations and ages of all surviving mature individuals. Recruitment dates were binned into decades to acknowledge uncertainty in exact ages. While we can reconstruct recruitment dates from the subset of individuals that survived to adulthood, we do not have data on seedling recruitment pulses or survival rates of young trees, which have been shown to play a role in other expanding limber pine populations (Millar et al. 2015). At five of the six populations, every individual tree was surveyed. One population, PF_ANCHOR_, had more limited sampling than the others (see Table S1 for location and size of each population), where a new ranch owner forbade fieldwork on their land. The unsurveyed area at PF_ANCHOR_ comprised ~50% of the habitat area, and ~40% of the population.

No dead adult trees were found in three of the six populations (PP_CASTLE_, PP_COTTON_, PP_GRASS_), and four dead trees (1.5% of the population) were found at PF_CASTLE_. 8 out of 397 trees (2% of the population) at PF_ANCHOR_ died before 1970. 38 additional dead trees were found but unidentified to species, belonging to either PP_ANCHOR_ or PF_ANCHOR_. All dead trees were excluded from analyses because of uncertainty in dating years of establishment and death. Large numbers of already dead mature trees that are now undetectable are unlikely given the aridity of the landscape, nor did we detect evidence of beetle kill, but we added a “time since population initiation” effect to check for any evidence of disappearing adults in preliminary analyses. These models using adult numbers as predictors are estimating per capita reproduction and establishment. Recruitment should be underestimated in earlier years if there is cryptic mortality, so a “time” variable with a negative coefficient estimate would suggest a “ghost of trees past” effect. The coefficients for a time variable for top population models ranged from 1.57 to 4.28, so we concluded that cryptic early mortality is unlikely, and excluded the variable from further analyses.

### 2.2 Climate Data

We obtained estimates of reconstructed historical precipitation data from Cook et al. (2008), which are given as annual estimates for 10° × 10° (lat/long) grid points across North America from years 0 to 2006 CE. We obtained reconstructed historical surface air temperature data from Trouet et al. (2014), which span 1470–1970 CE and utilize pollen record data for reconstruction. We averaged estimates over 10‐year increments to obtain decadal temperature and precipitation values for our analyses. These two datasets were chosen because they provided the smallest grid size and because the reconstructions encompassed the entire time span for our population data. Annual temperature and precipitation data from these sources are the same for all of our populations, as all populations fall within the same grid cell. We truncated the population and climate data to not include information after 1970, as this means we had both complete single‐source precipitation and temperature data, and because our data—which span from the year 1470 onwards—is only a record of trees that survived to maturity (at least 50 years old; see Methods for detail). Inclusion of data from establishment events post‐1970 would introduce a subset of data on young trees that may not survive to maturity and are thus not comparable to the sample of trees in the more distant past.

## 3 Methods—Modeling

We use a combination of modeling approaches to disentangle the effect of different forcings on population growth, each of which are outlined below in greater detail, but summarized here first to provide an overview: First, we use generalized linear models to fit predictive models of population growth through time for each population (site), and for each species together with site as a fixed effect. To judge the relative importance of different predictors, we ran each of these models excluding either climate predictors, density effects, or fixed effects of site. To exclude density effects, we needed to use MCMC methods, fit in JAGS, to force the density effect to be a constant function of the number of adult trees. Next, we used point pattern analyses to look for evidence of facilitative or competitive effects where trees established relative to neighbors. We constructed a measure that we refer to as “effective area” which estimates the amount of available suitable habitat for establishment to see whether such a measure is a more useful predictor of new recruits than density alone. Last, we used the top predictive models from our first steps to run simulations reconstructing the population dynamics for each population to investigate the role of demographic stochasticity and the predictive accuracy when different classes of effects are left out of the fitted models.

### 3.1 Predictive Models of Establishment—Population Models

Separately for each population, we fit a series of alternative negative binomial models (function glm.nb, package MASS in R, Ripley et al. 2024) with a log link function to predict the number of recruits established per decade. All models included the number of mature trees in the decade of establishment as a predictor. Trees were considered mature after they reached 50 years of age, which is roughly the time that both species begin to produce substantial numbers of cones with viable seeds (Johnson 2001). Though *P. ponderosa* can bear cones as early as 7 years of age, most viable seeds are produced from trees 60 or older (Oliver and Ryker 1990). *P. flexilis* also begins cone production around 50 years. To test whether a 50‐year criteria was reasonable, we re‐ran top models to test the effect of using different estimates for maturity ranging from 10 to 100 years. In two of the six populations, using 50 years as a cutoff for mature trees was the best (judged by AIC_c_ and *R* ^2^ values). For the other four populations, the models with a higher cutoff estimate had the lowest AIC_c_ (between 70 and 100 years). However, delta AIC_c_ values between models with the best and a 50‐year criteria were < 2 in all but one case. In those four populations, the differences in pseudo *r* ^2^ values were also very small (0.003–0.07) so we used 50 as a consistent cutoff for all populations.

To construct models that allow a clear ecological interpretation, we included the number of mature trees (MT) and the natural logarithm of the number of mature trees (ln(MT)) into the linear predictor function. With a log link function all explanatory variables are exponentiated, so ln(MT) represents linear scaling of recruits to mature trees if its coefficient is ~1, while a coefficient less than or greater than 1 indicates negative or positive density dependence, respectively. The unlogged density term, MT, is exponentiated like all other predictive terms in the model and adds more flexible negative density dependence, such that with higher MT, the model can predict declining recruits (as opposed to just slower increases). With the log link function and a coefficient for ln(MT) = 1, these two terms result in a Ricker model growth function for recruit production (see Appendix A for more detail). It is also a more flexible and biologically interpretable functional form than is a quadratic relationship.

In all models, possible climate variables included the average annual precipitation and temperature values for the establishment decade and each of the two decades following (referred to below as the first, second, and third decade), reflecting the need for favorable conditions for young trees to survive several decades to establish and later be seen in our 21st century surveys. We ran all possible combinations of terms in these fairly simple models with *dredge* (package MuMin in R, Barton 2023) and tabulate results for all models that in sum include 90% of the AIC_c_ weights for each population. Only the top model in each category was used for subsequent population simulations. To test the predictive importance of different classes of effects, we also identified the best fit models that did not include any climate effects and the best ones that omitted nonlinear density effects.

To estimate models that did not include nonlinear density effects, we used JAGS (packages *rjags* and *runjags* in R, Denwood and Plummer 2023, Plummer, Stukalov, and Denwood 2023) so that we could most easily constrain the coefficient of *ln*(MT) to one. We note that the coefficient estimates from *glm.nb* and our unconstrained JAGS models were nearly equivalent. We used the *dic* function in *rjags* to judge JAGS model fit. We refer to all these separately fit population models as “population models.” See Supporting Information methods for more detail on JAGS models.

### 3.2 Predictive Models of Establishment—Species‐Wide Models

To judge how divergent the effects of various factors were for each population, we also constructed models for all populations of each species with all terms described above plus two‐way interactions between populations as a fixed categorical effect and each other term in the model (climate variables and number of mature trees). Again we used *dredge* to find the top models (judged by AIC_c_) given all possible combinations of terms described above. We refer to these as “species‐wide” models and contrast their results with the best species‐wide models that eliminate either climate, density dependence (using JAGS models), or site effects.

To summarize: for each of the six populations, possible explanatory variables included the number of mature trees (MT) and its natural logarithm (ln(MT)), climate variables (first, second, and third decade post‐establishment temperature and precipitation data), and interactions between density and climate variables. These are referred to as “population models.” We then pooled each species' data and added a fixed effect of “site” as a possible explanatory variable, climate and density variables described above, and possible interactions between site and density variables with climate. These models are referred to as “species‐wide” models.

### 3.3 Spatial Analyses

Given that trees are stationary, overall population numbers may be less informative about density than local scales, so we also conducted analyses to examine spatial patterning during establishment. These analyses were used both to test for the need to incorporate fine‐scale spatial information into our other results, as well as to better understand the interplay between positive and negative density dependence in recruitment dynamics. First, we looked for trends in how closely recruits established mature trees through time. We used pair‐join analyses (also called pair‐correlation analyses) to determine the regularity of spatial pattern at different spatial scales to test for positive (i.e., facilitative interactions) or negative (i.e., competitive) association (Diggle 2014). Negative density dependence at these smaller scales would manifest as overdispersion in establishment locations, such that there are fewer than expected recruits establishing close to existing trees. Though these analyses cannot disentangle the exact nature of small‐scale interactions, facilitative effects could include clustering of high‐quality patches for seedling establishment, nurse object availability, or mycorrhizal associations (Brodersen et al. 2019, Marsh, Blankinship, and Hurteau 2023, Ouzts et al. 2015), which have been shown to increase seedling survival in other systems.

Then, we constructed a measure we refer to as “effective area,” which is a proxy for the amount of habitat available for establishment in each decade for each population. Effective area is the amount of suitable, unoccupied area likely to support recruits; it is a measure that considers both total available area within the delineated habitat boundaries and weights that area by how likely establishment is at different distances from existing mature trees. We established this weighting function by first dividing the landscape area into 0.5 m × 0.5 m pixels. We calculated the probability density of distances from each available (unoccupied) pixel to each tree on the landscape for each decade, separately for each species. We then weighted this density kernel by multiplying the magnitude of the probability density function value of these distances by the density of observed establishment distances.

Effective area tends to increase as the number of mature trees increases, but then can level off as there is less area suitably far from established trees (Figure S4). Areas proximate to adults may be more suitable for establishment because adults provide shade, mycorrhizae networks, or simply because successful individuals have survived and grown in that beneficial microhabitat for other reasons which may also be amenable to new recruits. To test whether effective area is a markedly better measure of local density effects, including negative density dependence, that is number of mature trees, we replaced mature adults with effective area in our establishment models and compared their predictive power. Detailed methods and results of our spatial analyses are in supplemental Appendix A. While these spatial analyses give insight into interactions between trees, we did not find that use of effective area significantly improved the predictive accuracy of establishment models over the use of total mature trees, nor did it alter the general results we find using mature tree numbers rather than effective area. Therefore, we do not present simulation model results based on these spatial models in the main text (see Figure S6, Tables S13–S20 for EA results).

### 3.4 Stochastic Simulations

To compare the predictive accuracy of models that use or omit different classes of effects, we used each of the classes of models described above (the top models for population and species‐wide models, and those eliminating different classes of effects) to simulate the age distribution and size of pine populations over time. We initiated each simulation with the first year that a single tree became mature in each population, also including any existing juvenile trees present at that time. We then used decadal climate data and the simulated number of reproductive trees to predict recruitment through time; in each year we generated a number of recruits based on this mean (and the model‐predicted dispersion, which is the inverse of variance) using a negative binomial random number generator (R function *rnbinom*). We constrained the *rnbinom* estimate to be no > 3× the mean recruitment estimate (this corresponded to at least the 0.92 quantile value for a negative binomial, and generally > 0.98 quantile value across all estimates per population per year). This procedure incorporates the effects of demographic stochasticity on recruitment. We ran the simulation 500 times for each population and model type. To remove the effect of demographic stochasticity, we also generated predictions using the non‐integer estimates of mean recruits per decade, iterating the number of average expected recruits per decade into the future. To further examine the effects of increased demographic stochasticity and altered dynamics at the very low numbers seen early in establishment, we also ran our base simulations starting in the first decade when five mature trees were present to see how well the model simulations could predict dynamics if we eliminated the initial periods of very low numbers and often slow population growth.

We took two approaches to compare the predictive accuracy of these simulations. First, we used the proportional deviation of each decades' median model prediction from the observed number of trees. Second, we determined the fraction of decades that each population's true numbers were outside the 90% simulation envelope of a given model's predictions.

All analyses were run using R version 4.3.2 (R Core Team 2023).

## 4 Results

### 4.1 Establishment patterns across time and space

**FIGURE 1:**
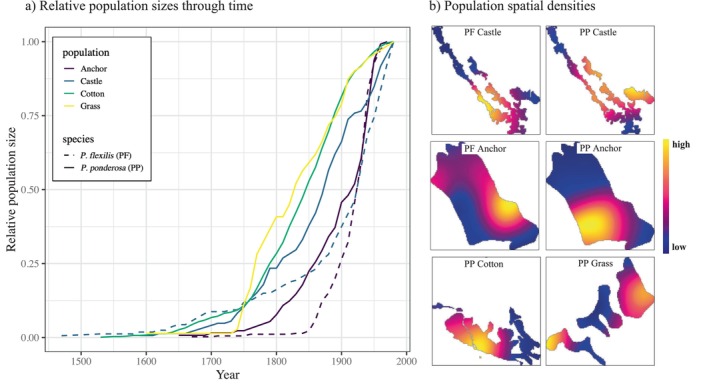
(a) Relative population sizes from population initiation until 1970. Curves show total population sizes, including juvenile and mature trees. (b) Local spatial arrangements of each population. See main text for habitat delineation. Scale bar from high to low density is relative to the maximum density value in each panel.

All populations experienced a long initial colonization period with very little recruitment, followed by increasing numbers until the present (Figure 1; figure 2 in Lesser and Jackson 2012). In spite of these similarities, the populations did not show highly correlated growth and also varied substantially in their recruitment rates across time (Figure S1, Table S2). The two pairs of co‐occurring species also showed substantially different patterns of growth (Figure 1), suggesting that shared external drivers and local habitat features affected each species differently. We saw no evidence of masting events in the recruitment rate seen in these populations.

**FIGURE 2:**
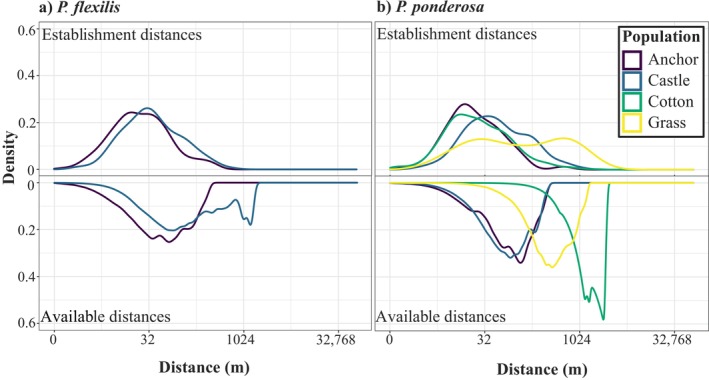
Density distributions of establishment distances to the nearest mature tree at the time of establishment (shown above the *x*‐axis) versus distributions of distances to the nearest mature tree from each point within the entire defined landscape area (available distances, shown below the *x*‐axis) for each population. Note the log2 scaling. Recruitment is markedly higher close to mature trees than far away, but few recruits establish extremely close to established individuals. See text for details on landscape delineation. PF populations shown in left panel and PP populations on the right. The density of available distances is shown for 1970, and all trees that recruited prior to 1970 are included in the density of establishment distances.

Density functions of establishment distances show that recruits disproportionately establish close to mature trees when compared to all available suitable habitat (Figure 2). However, recruits rarely established extremely close to adults; the nearest recruit was virtually always > 2 m away and the majority (66%) of establishment distances were between 5 and 100 m from the nearest mature tree, suggesting that either dispersal limitation, positive density dependence, perhaps due to mycorrhizal relations (Habte, Miyasaka, and Matsuyama 2001), or both can limit establishment on the far side, and competitive or exclusionary effects may limit recruitment at close distances to mature trees. Other small‐scale microhabitat effects, such as aspect, soil conditions, and shade availability likely also influence spatial patterns of establishment. The species were similar in modal establishment distance, with PP at 11 m, and PF at 9 m. The 95th percentile establishment distances were 204 m for PF and 303 m for PP.

Quantile regressions show that establishment distances decreased through time for most populations (Figure S2). However, the majority of these decreases were due to reductions in the long tail of establishment distances. The 80th percentile establishment distances decreased most rapidly, presumably because the maximum possible establishment distance becomes constrained by the number and distribution of mature trees as they fill in the limited suitable habitat at each site. In contrast, the 50th (median) and the 20th percentile predictions did not vary strongly over time, suggesting relatively constant close‐range establishment constraints even as populations grew.

### 4.2 Population models— *P. ponderosa*

**FIGURE 3:**
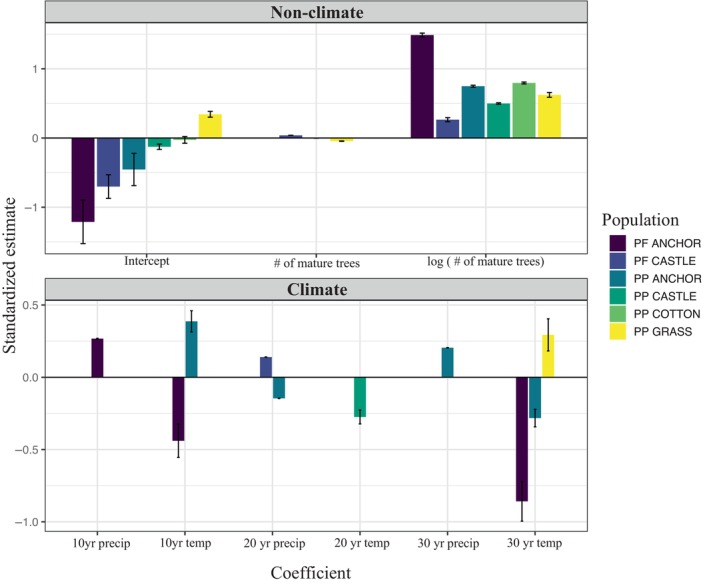
Standardized coefficient estimates and SD for all top population models. Non‐climate variables in top panel, and climate variables are shown in the bottom panel. Climate variables are annual averages (i.e., 20 year. temp temperature in second decade post‐establishment).

All four *P. ponderosa* population models show support for density dependence on establishment numbers through time, where the coefficient of ln(MT) < 1 in all top models, and negative effects of MT in PP_COTTON_ and PP_GRASS_ models (Table 1; Tables S3–S6). Support for climate variables was mixed across populations (Table 1). PP_COTTON_ showed support for zero or one climate variable in all top models, PP_GRASS_ and PP_CASTLE_ models most often included one climate variable (but a minority included zero or two), and top PP_ANCHOR_ models generally showed support for four climate variables (Tables S3–S6). Overall, there was support for some combination of temperature and precipitation variables, but with different decadal climate variables supported for different populations, and inconsistent coefficient signs for both precipitation and temperature both within and across population models (Figure 3). In spite of these differences between populations, models that included climate were generally supported over no climate models for three of the four populations, with delta AIC_c_ values of 0.846–8.64 between the best model and the best no‐climate model. Similarly, models that included nonlinear density dependence (coefficient of ln(MT) ≠ 1) were supported over models in which the coefficient of ln(MT) = 1, with delta DIC values of 3.08–28.44 (Table S23). For all four populations, the fitted coefficient estimate was < 1 (Table S11), indicating nonlinearly declining ratios of recruits to mature trees over time (negative density dependence).

**TABLE 1:** Top models of establishment for each population judged by AIC_c_.

Population	Intercept	# mature trees (MT)	ln(MT)	1st decade precip	1st decade temp	2nd decade precip	2nd decade temp	3rd decade precip	3rd decade temp	df	Weight
PP_CASTLE_	−0.127		0.499				−0.275			4	0.0546
PP_ANCHOR_	−0.454		0.748		0.387	−0.146		0.204	−0.282	7	0.1238
PP_COTTON_	−0.027	−0.003	0.795							4	0.0245
PP_GRASS_	−0.342	−0.048	0.623						0.293	5	0.0413
PP_CASTLE_	−0.701	0.038	0.264			0.140				5	0.0278
PP_ANCHOR_	−1.212		1.488	0.267	−0.439				−0.858	6	0.0272

*Note:* See text for descriptions of possible predictor variables.

### 4.3 Population Models—*P. flexilis*

Both population models showed positive effects of ln(MT), while PF_CASTLE_ models also showed support for a positive MT effect (Tables S7 and S8). The two PF populations showed support for climate effects, with top models including 1–4 climate variables, with the exception of one of the top models at PF_CASTLE_ which included no climate effects. Precipitation effects were mostly positive, and temperature effects mostly negative. The top models that included climate were again supported over no climate models, with delta AIC_c_ values of 0.27 (PF _CASTLE_) and 15.15 (PF _ANCHOR_) between the best model and best no‐climate model. At PF_CASTLE_, models without density were marginally supported (delta DIC = 0.99), and the coefficient estimate was < 1, indicating negative density dependence. At PF_ANCHOR_ models with density were strongly supported (delta DIC = 10.73), and the coefficient estimate was > 1, indicating positive density dependence, or an increasing ratio of recruits to mature trees through time.

### 4.4 Population Models—Stochastic simulations

**FIGURE 4:**
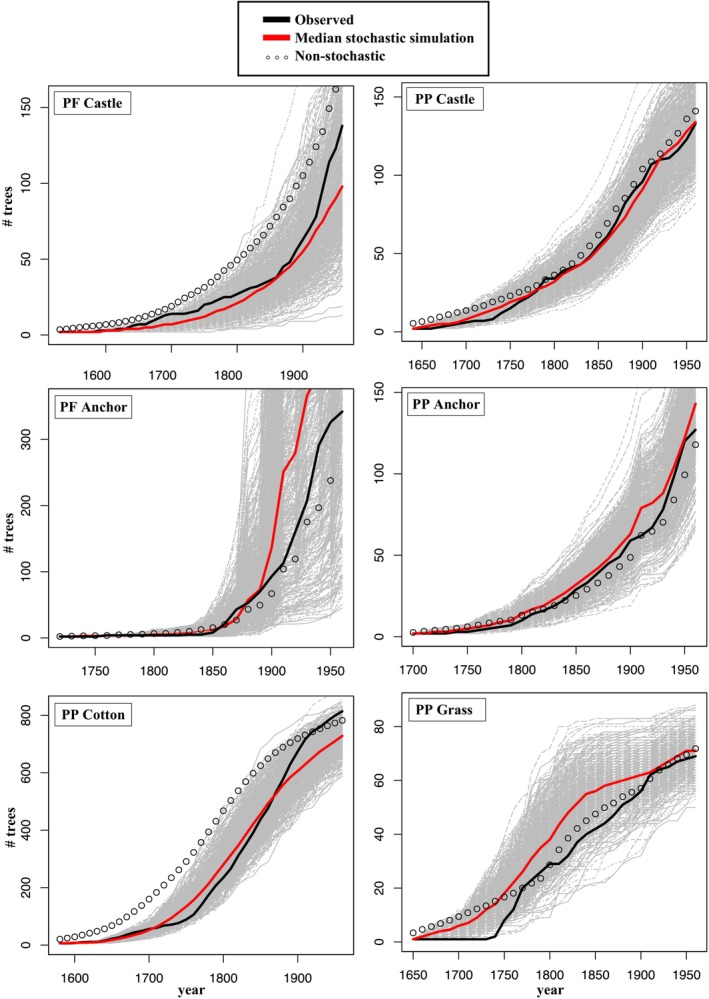
Results of stochastic simulations for the best‐supported population model (our “population models,” Tables S3–S8). Plots show the number of mature trees at each time step. Simulation results are from 500 runs. Note that we ended the simulations at 1950 because our complete climate data only extend to 1970, and predictor variables for some populations included third‐decade post‐establishment climate data. See Methods for details. The median value of all stochastic simulations is shown in red. The non‐stochastic simulation is dotted, and actual population numbers are in black. Gray lines show the results of individual runs.

Simulations using the best‐supported model for each population yield a fairly broad range of predicted numbers, but the median predicted size in each year generally tracked actual population numbers well (Figure 4). Notably, stochastic simulations produced much broader ranges of estimates for *Pinus flexilis* populations. Though many simulations tended to either consistently under or overpredict population growth for a given population, the predictions of actual population numbers were generally well matched by the median simulation estimates.

**FIGURE 5:**
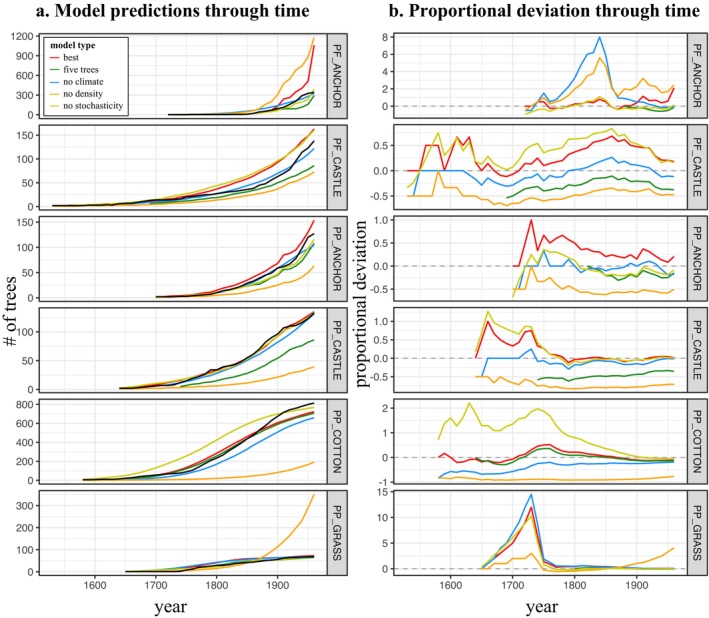
(a) Median predictions of all classes of stochastic population models. Actual data shown in black, with median of simulations using the best model for each population shown in red, using the top model initiated after five trees were present shown in green, the top model without climate variables in blue, without density in orange, and non‐stochastic predictions shown in yellow. (b) Annual proportional deviations ([model predicted total number of trees − observed]/observed) for each type of model.

Omission of stochasticity substantially degraded predictive accuracy by over‐estimating population growth at PP_COTTON_ and PF_CASTLE_ (Figure 4), and omission of density effects led to a underestimation of growth for most populations (Figure 5b). Omitting density dependence increased the mean proportional deviation from observed numbers by 30%–284% over that for the best models (Figure 5b).

Population models deviated more dramatically from omission of density variables than from climate variables, but removal of climate effects nonetheless increased mean deviations from those of the top overall model by 1.40%–67%, with the exception of PP_COTTON_ where no climate variables were included in the top model (Figure 5).

**FIGURE 6:**
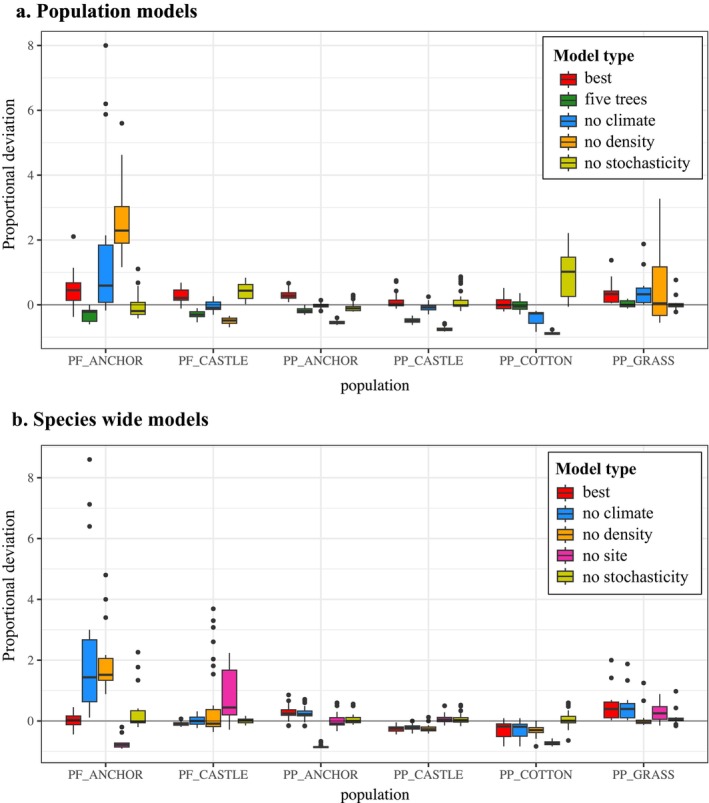
Boxplots of proportional deviations from the top model predictions after 5 adult trees were present to actual population numbers for (a) population models and (b) species‐wide models. Deviations from median stochastic simulations that used best population model (red), best model fit with only data from when 5 adult trees were present in each population for population models (green), top model without climate variables (blue), without density variables (orange), non‐stochastic model (yellow), and species‐wide models without a fixed site effect (pink).

For some populations, there was virtually no effect of omitting the randomness inherent in recruitment events (e.g., PP_COTTON_, PF_ANCHOR_), but for the others, non‐stochastic models resulted in substantial overestimation in the first century or so (e.g., PP_GRASS_) (Figure 4). However, most proportional deviations after excluding early years with < 5 trees more closely tracked population growth (Figures 5b and 6a). Models that did not use the earliest, lowest‐density data performed extremely well, better than the median predictions of the best fit model using all data with the exception of PF_ANCHOR_ (Figure S5). This improvement is likely due to the omission of data when the populations were most influenced by demographic stochasticity and possibly pollen limitation.

In the same population, the same class of effect can have different importance through time (Figure 5). At PP_GRASS_, for example, while all models performed worse in the first century post‐colonization than the second, exclusion of climate or stochasticity initially led to larger deviations than density effects, which mattered far more for predicting dynamics in the second century. Non‐stochastic models tended to perform worse in the first hundred years for each population, while density and climate effects tended to matter more for accurately predicting dynamics as population sizes increased (Figures 5 and 6a). At all sites, proportional deviations for models that excluded different effects decreased through time, suggesting that larger population numbers may be more resilient to fluctuations in climate, stochastic variation, or density effects, as exclusion of any of these mattered more in the first century following colonization than most decades afterwards (Figure 5b).

To judge the consistency of causal effects across populations, we also reran the best model for each of the six populations to generate standardized coefficients for each included effect (Figure 3). These show that while density effects were quite consistent, the strength, identity, and direction of climatic factors included in the models were highly variable across populations (Figure 3).

### 4.5 Species‐Wide Models

Species‐wide models for PP showed strong and consistent support for # of mature trees, MT, and ln(MT), both positive, as well as the categorical population effect (Table 2 and Table S9). We also found strong support for interactions between population and MT, and support for a population by third decade temperature effect. Though no climate variables were present in the top model, all other models with AIC_c_ weight > 0.01 included one or two climate variables. We found no strong support for climate by population interactions, suggesting similar climate effects across populations, a result in conflict with the population‐specific models.

**TABLE 2:** Top models of establishment for each species with population (S) as a fixed effect.

Species	Intercept	*S*	MT	ln(MT)	IP	IT	2P	2T	3P	3T	MT*S	ln(MT)*S	1P*S	1T*S	2P*S	2T*S	3P*S	3T*S	df	Weight
*Pinus ponderosa* (PP)	−0.519	*	−0.004	0.820							*								10	0.0375
*Pinus flexilis* (PF)	−2.953	*	−0.049	2.714		−0.393				−0.761	*	*						*	10	0.0306

*Note:* MT = # mature trees, log(MT) = natural logarithm of # of mature trees. Climate variables abbreviated to indicate decade followed by variable type, e.g. IP = one decade post‐establishment precipitation average, 2T = two decade temperature average post‐establishment, etc.

* Inclusion of fixed effect, or interaction with fixed effect.

The PF species‐wide models included a negative effect of MT and a positive ln(MT) term, and all included a population term (Table S10). All top models also include an interaction between population and both density terms. The fitted coefficient estimate was 0.72 for PP, indicating declining numbers of recruits per adult tree through time (negative density dependence), and 1.73 for PF, indicating positive density dependence (Table S24). Models that included non‐proportional effects of mature trees (coefficient of ln(MT) ≠ 1) were always supported over models without these effects, with delta DIC values of 1.13 (PP) and 16.06 (PF).

The PF species‐wide models showed support for more climate variables compared to PP—all top models include first and third decade temperature (negative effects) and mixed support for first decade precipitation (positive effect). We also saw strong support for population by third decade temperature interaction and mixed support for interactions between population term and first decade temperature and precipitation. The top PP models show inconsistent support for climate effects and primarily included negative effects of second‐ or third‐decade temperature.

Finally, we tested the effects of site differences on predictive power by using our top species‐wide models to simulate growth and including/excluding population as a fixed effect along with any of its interactions. As with the single population models, relative importance of different classes of predictors substantially differed between populations. For example, removal of climate and density was most detrimental to model predictions at PF_ANCHOR_ while removal of site effects worsened PF_CASTLE_ predictions most severely (Figure 6b, Figure S3b).

Across the board, exclusion of site had a substantial influence on model predictions. The best‐supported simulations using species‐wide models without a fixed effect of site (or any site interaction terms) performed markedly worse than both the top species‐wide and top population models. Models without any site effects increased mean absolute errors by 10%–186% over top models (Table S22). Effects on model performance of excluding climate, density dependence, or stochasticity, while including site effects, were similar to those seen in the individual population models, with mostly strong effects from removal of any class of effects (Figure S3b, Table S22).

## 5 Discussion

Ecologists have recognized the importance of density dependence, climate, and stochasticity on population growth, but the relative importance of each has been subject to continued debate. Recently, the need to predict range shifts and population persistence—processes which require successful colonization and subsequent population growth—has been a focus of numerous studies, and most of this work heavily relies on past and projected climate data to predict future population dynamics. For example, widely used species distribution models (SDMs) generally use a species' climatic niche to project its future distribution without accounting for intrinsic drivers such as stochasticity, or intraspecific differences across populations (but see DeMarche, Doak, and Morris 2019, Chardon et al. 2020), and also hinge on reliable predictions of climate effects on population dynamics. Despite the spatially explicit nature of the range shift literature, few studies incorporate within‐population density effects or population dynamics into predictive models (DeMarche, Doak, and Morris 2017). Here, we test for the generalizability and relative importance of intrinsic and extrinsic drivers across space and time and find that successful prediction of dynamics relies on inclusion of climate, density, and stochasticity, with their relative importance varying in idiosyncratic ways between populations. Though the two species here vary in their climate tolerance across their broader range, they coexist in a narrow elevational band in our study area and are likely both limited by the severe, arid climate, and interspecific competition. Given their overlapping climate and habitat, as well as the relative proximity of the populations to one another, the variation in the drivers and resulting population dynamics was surprising, especially for long‐lived organisms that are often buffered against more transient environmental effects.

While all of our studied populations grew from a few initial recruits to substantial numbers over the study period, at a finer scale the population trajectories of our six focal populations shared few commonalities, with low correlations in establishment rates through time. Most populations, unsurprisingly, experienced several decades of low numbers and close to zero growth before a period of more rapid increase in numbers. However, the length of this early low‐growth period varied dramatically between populations; PF_ANCHOR_ persisted with < 5 individuals for nearly 200 years after colonization, while other populations broke through this small number barrier after four or five decades. Pollen limitation and lack of available seed, as well as local inbreeding may have prevented early growth until enough individuals dispersed into the region. Lesser and Jackson 2013 showed that age at first reproduction decreased through time, demonstrating that age and/or tree size was not inhibiting successful reproduction during the early decades following population establishment, strengthening the argument that in these early years, inbreeding and/or Allee effects may play a strong role in limiting reproduction (in conjunction with more favorable climate in later years, which also may have increased growth rates and decreased age at first reproduction). Notably, important seed dispersers (i.e., *N. columbiana* ) are increasingly likely to visit populations as the abundance and reliability of seed set increases; thus, once these populations reached a critical size, local and foreign seed dispersal volume may also have increased and helped overcome early genetic constraints. Future work could consider genetic diversity within and between populations to assess whether genetic variation is constraining population growth in these isolated areas.

After periods of roughly exponential growth, increases in four of the six populations have slowed, with final population numbers ranging from ~70 to 800 individuals. The final density of each population was also quite different, ranging from ~4 to 50 trees per km^2^ (Figure S10), though it should be noted that this is partially an artifact of the way we delineated habitat; some populations have yet to colonize portions of supposed available habitat. In addition, the habitat areas are topographically complex and may differ in the fraction of total area that provides suitable establishment sites in ways we could not quantify.

This lack of temporal correlation in growth dynamics contrasts with the very similar spatial structuring within populations. Pair‐join analyses show strong clustering of individuals across a range of spatial scales both within and between species, where both species were censused, as well as between recruits and mature trees (Figures S7 and S8). At all populations, we found evidence of clustering but very little dis‐association, suggesting that at both historical and current numbers, positive density dependence and/or dispersal limitation has played a role in establishment distances, as well as number of establishment events. Recruits most often established very close to mature trees, though they are able to disperse much further. This suggests either that existing adults occupied the most favorable microsites or that proximity to adults offers shelter or perhaps shared microbial networks (Habte, Miyasaka, and Matsuyama 2001), and not a lack of dispersal ability. Despite positive association of recruits to mature trees, recruits rarely established closer than 2 m from the nearest mature tree, likely due to direct resource competition of closely adjacent individuals.

We also found strong support for the role of climate on establishment and population growth. Temperature effects were somewhat inconsistent between populations—sometimes influencing recruitment positively and sometimes negatively. Even within populations, temperatures in the first decade after establishment versus the second or third decade had different effects, suggesting that optimal conditions for recruitment may not be the same for early survival and growth in the subsequent two decades and that optimal conditions varied between populations and species. Precipitation variables were similarly variable, though they more often had positive effects. Despite this variation, comparison of top models with and without climate variables shows that climate played a substantial role in shaping dynamics in five of the six populations. While some of these effects may be driven by local site characteristics, we note that models for PF_ANCHOR_ and PP_ANCHOR_, which share the same site, showed support for different climate variables as well as shared climate variables with opposite effects. Similarly, the two Castle populations showed support for different climate variables. Together, these results suggest that even in shared conditions at smaller scales, the two species differ in their necessary recruitment conditions. The temporal resolution of the available climate reconstructions is not fine enough to capture rare, severe climate events, or extreme conditions that limit seedling survival or contribute to juvenile mortality, but even the relatively coarse reconstructions do indicate that climate is a meaningful driver of population growth in this system. Notably, differential responses between similar populations are not attributable to any obvious slope angle, aspect, soil composition, or elevational differences, though there is some spatial separation between species at shared sites (Figure S11).

Like climate, density dependence was necessary to produce models with reasonable predictive accuracy for most populations The loss of predictive power from removing density effects was large for most populations, and unsurprisingly, generally had a larger effect as population size increased (Figure 5). All top population models included a positive linear density effect (ln(MT)), though the coefficient value ranged from 0.26 (PF_CASTLE_) to 1.49 (PF_ANCHOR_). All populations except PF_ANCHOR_ showed signs of declining numbers of recruits per adult over time (negative density dependence; Table S11). This could be due to unaccounted for effects of mature trees in the uncensused habitat area for this population. The nonlinear density term (MT) was consistently supported in only three of the six top population models. This term was negative for PP_GRASS_ and PP_COTTON_, suggesting negative density dependence, and positive at PF_CASTLE_, suggesting continuing and nonlinear positive density dependence (Table S11).

The two PF populations studied reached similar densities to (and co‐occur with) the PP populations, and the ecology of the two species is similar. Given this, we also tested for overall density effects by including a term in our models for the total number of trees of both species in sites where both populations were censused. These models had far less predictive power than species‐specific density alone. This result, together with low correlations in growth in the two pairs of populations that share the same site, suggests that the presence of coexisting species plays little role in altering population dynamics for these populations.

Site effects were also important for both species; species‐wide models that tried to predict general effects of density and climate, even with a fixed effect of site, led to considerably worse predictions of population trajectories than our population models. On one hand, this is hardly surprising, but most efforts to project future numbers or population spread do so from limited data on one or a handful of existing populations. This is particularly worrisome, as the effects of ignoring site were among the largest effects we saw (Figure S3). Careful characterization of cryptic microhabitat effects that are site‐specific might offer more concrete explanations for differential responses of populations to shared climate drivers. In particular, a study investigating microhabitat characteristics that might influence both recruitment and survival rates might help explain the mechanisms behind small‐scale spatial patterns in recruitment and larger‐scale population dynamics.

The role of demographic stochasticity in shaping each population's dynamics was investigated by comparing stochastic and non‐stochastic simulations. Though the 90% confidence intervals generated by stochastic simulations produced a wide range of ending population numbers, especially for PF populations, the median of these stochastic simulations generally looked similar to the non‐stochastic simulations (Figure 4). Median stochastic estimates performed similarly to non‐stochastic estimates and were most often over‐ or under‐predicting actual population numbers in the same direction. The envelope containing the 500 stochastic simulations contained the observed population trajectories for five of our six populations throughout the population history, and the sixth (PP_GRASS_) was within the interval for the ~2/3 of its history, including the latest periods. In a different test of how demographic stochasticity can influence the process of fitting and predicting dynamics, we saw much‐improved predictions when we excluded early years with fewer than five mature trees present. This implies that while the earliest period of population growth and establishment are crucial for eventual numbers and dynamics, they may be extremely difficult to predict in practice. This may be due both to the greater effects of demographic stochasticity in small populations, as well as the likelihood that different processes are present at low numbers (Lande 1993; Kelly et al. 2021).

Notably, early lags in population growth at all populations were difficult to predict and varied widely, with some populations breaking through this early barrier relatively quickly (< 50 years) and others doing so much more slowly (> 150 years). This variation is unlikely to be driven by diverging climate effects, since all the populations occur within the same general biogeographic and climatic region. Though we were able to predict population dynamics well after five trees established, that still leaves decades or even centuries of uncertainty, which is a large window given the velocity of climate and land use change in many areas. This pattern very likely exists for many colonizing populations, which could make accurate prediction of new population growth or range expansions difficult.

Taken together, density, climate, and site effects collectively were able to predict population growth relatively accurately, especially when initiating models only after five mature trees were present. Removal of any one of these effects worsened model predictions to varying degrees for each population. We do not find that any one class of effect had a predominant impact or that any could generally be ignored. The two species we examined responded to shared effects in unequal and even opposing ways, despite occupying the same landscape with shared climate and density effects. In some sense, this conclusion is a surprise for our populations. All are relatively sparse, and their growth trajectories do not show striking effects of density dependence. Furthermore, many demographic studies of similarly long‐lived, slowly growing populations have ignored one or more of the factors we address here, especially density dependence and demographic stochasticity. One might suspect that climate is the chief driver of long‐term population dynamics, but our analyses suggest that interspecific interactions and spatial and temporal density effects are equally vital to accurate forecasts. This work suggests that greater care be taken to address these complications when possible and caution in creating predictions when we are unable to estimate these effects.

## Author Contributions

**Ellen Waddle:** formal analysis (equal), investigation (equal), writing – original draft (lead), writing – review and editing (lead). **Daniel F. Doak:** conceptualization (equal), formal analysis (equal), funding acquisition (equal), methodology (equal), project administration (equal), supervision (equal), writing – original draft (equal), writing – review and editing (equal). **Christopher Steenbock:** data curation (lead), formal analysis (supporting), investigation (supporting), methodology (supporting). **Mark R. Lesser:** conceptualization (equal), data curation (equal), formal analysis (supporting), investigation (lead), methodology (lead), project administration (equal), writing – review and editing (supporting).

## Conflicts of Interest

The authors declare no conflicts of interest.

## Supporting information


Appendix S1

## Data Availability

All of the data and required code for stochastic simulations and JAGS models with constrained density coefficients are included in Supporting Information.
