# Supplementary material for: Density, Climate, and Stochasticity Shape Four Centuries of Population Dynamics for Two Long‐Lived Tree Species
Source: Ecol Evol. 2024 Dec 15;14(12):e70664. doi: 10.1002/ece3.70664 (PMC11646622; doi:10.1002/ece3.70664)
Supplement: Supplementary file 1 — Appendix S1 [file ECE3-14-e70664-s001.zip › READme.docx]

- *bighorn_dat.csv*: full data file of ages and spatial locations of all censused trees
- *data files for simulations:* contains 8 data files (6 ‘population’ data sets, 2 ‘species-wide’ data sets) with climate reconstruction data added in to be used in stochastic simulations
  - *example file name: ‘anchor_pifl_simulation_input.csv’*
- *stoch_simulations.R*: code used to run stochastic simulations using best model for each population
- *run JAGS models.R*: code to run JAGS models, which are all provided in “JAGS models” folder with file names written as ‘*PFanchor_noDensity_JAGS.R’*
