# Supplementary material for: Density, Climate, and Stochasticity Shape Four Centuries of Population Dynamics for Two Long‐Lived Tree Species
Source: Ecol Evol. 2024 Dec 15;14(12):e70664. doi: 10.1002/ece3.70664 (PMC11646622; doi:10.1002/ece3.70664)
Supplement: Supplementary file 1 — Appendix S1 [file ECE3-14-e70664-s001.zip › Supplemental Material.docx]

**SUPPORTING INFORMATION**

- **Supplemental Methods – Appendix A**
  - Available habitat delineation
  - Pair-join analyses
  - Effective area analyses
  - Effective area results
  - JAGS Models without negative density dependence
- **Supplemental Figures**
  - **S1**: Lambda vs population size through time
  - **S2**: Quantile regressions of establishment distance vs time
  - **S3**: Median predictions of spp-wide models and proportional deviations thru time
  - **S4**: Effective area vs # of mature trees
  - **S5**: Stochastic simulations initiated after five trees matured
  - **S6**: Stochastic simulations using EA
  - **S7**: Pair correlation function plots (I)
  - **S8**: Pair correlation function plots (II)
  - **S9**: Map of site locations
  - **S10**: Density of each population through time
  - **S11**: Distribution of trees in overlapping populations
- **Supplemental Tables**
  - **Table S1:** Location and size of the populations
  - **Table S2:** Correlations in two measures of growth
  - **Tables S3– S8:** Top population models
  - **Tables S9 – S10:** Top species wide models
  - Table S11: coefficient values for density in top population models
  - **Table S12:** Coefficients of models with ‘Time’ variable added
  - **Tables S13-18:** Top population models using effective area (EA)
  - **Tables S19- S20:** Top species-wide models using effective area (EA)
  - **Table S21:** Model deviations of top population models
  - **Table S22**: Model deviations of top species wide models
  - **Tables S23-**24: Top models with and without density effects
  - **Table S25:** Correlation matrix of climate variables used
- **References**

SUPPLEMENTAL METHODS

**Available habitat delineation** - All four population areas occur on discrete habitats bounded by apparently unsuitable habitat, largely dominated by sagebrush steppe communities. Areas of pine establishment are often rocky and include steep bluffs and slopes, while surrounding areas are lower. Pines commonly co-occur with *Juniperus osteosperma* and *J. scopularum*. We mapped polygons to delineate the border of suitable habitat for establishment in Google Earth Pro for each population. These 2D polygons included both occupied area and apparently suitable habitat adjacent to the population. Rocky outcroppings and dry *Juniperus* dominated areas vs. sagebrush habitat generally made suitable habitat boundaries apparent from these aerial images.

**Pair-join function analyses -**

*Assessing spatial associations of adults and recruits.* Localized density effects for sessile species are necessarily reflected in spatial associations, either negative or positive, and thus before modeling the temporal dynamics of our populations, we examined the spatial structure of each one over time. To begin, we used a spatial point process approach to test for non-random association between trees (Diggle 2014). The pair join function (Function *pcf* in the spatstat package in R, Baddeley and Turner 2005) can be used to test non-random associations between individual trees at different distances from one another. The pair join function has also been called the pair correlation function or Wiegand’s O in the ecological literature (Wiegand and Moloney 2004), and is similar to Ripley’s K, but is defined for annulae (a region bounded by two concentric circles) of distances from focal individuals, vs. Ripley’s K, which tests for non-random association for all individuals within a maximum radius from focal individuals (Baddeley et al 2014). By examining patterns using the pair join function, we can test for non-random densities (either positive or negative) at a range of distances from each individual tree. We expect that positive density dependence would show strong association (clustering) of individuals, and that negative density dependence would show more dis-association (overdispersion) than would be expected by random. Clustering of individuals might be found if recruits tend to do well if they establish nearby mature trees (even if they aren’t the offspring), perhaps due to shared mycorrhizal networks; another explanation for clustering of individuals (possibly of different species) can be that small microsite differences favor establishment and growth in some areas over others. Dispersal limitation could also result in clustering of recruits around parents of the same species. Disassociation would be shown if individuals compete for sufficient light or soil nutrients, or if seed predation is higher near mature trees.

At each site and for each species separately, we ran these analyses on three time periods: at the first decade when at least 10 mature trees were present (total population numbers ranging from 14-113), again when population numbers were about half of final number, and again at 1980. In addition to testing for non-random associations between all individuals, we looked for associations between mature trees and recruits. We also used function *pcfcross* (Baddeley and Turner 2005) to assess the spatial effect of one species on another, as well as the adults of one species on recruits of the other species. For all these analyses, we defined mature trees as those of > 50 years in age and recruits as those < 50 yrs (see main text for more cutoff decision description). Though ponderosa can bear cones as early as 7 years of age, most viable seeds are produced from trees 60 years or older (Oliver and Ryker 1990). Limber pines also begin cone production around 50 years of age (COSEWIC 2014). Shifting this cutoff to higher ages did not substantially influence the results (results not shown).

For pair-join analysis, the radii defining each annulus must be specified: because of the skew in inter-individual distances, we used 1m spacing for distances from 0-50m, and 10m spacing for 50-600m. To construct confidence limits around random expectations, we generated 500 random point patterns within the available habitat area of a population and applied the pair-join function to each of those patterns, using the 5^th^ and 95^th^ percentiles at each distance value to create a “simulation envelope” defining 90% of observations (cutoffs using stricter percentiles - 1^st^ and 99^th^ - showed qualitatively the same patterns). Thus, significance is assessed by comparing likelihood of spatial clustering relative to a purely random point process. To display these results, we transform the pair-join value to express the observed density as a proportion of that expected under complete spatial randomness Values of the transformed variable > 1 indicate clustering while values < 1 indicate dis-association. Note that density dependence at these scales may operate in tandem with, or independently of, population-level density dependence, which we test for in later analyses. For example, at small scales, new recruits may be spaced farther from existing adults than expected, but the population may still be growing exponentially with no signal of population-level NDD.

**Pair join test results -**

*Same species.* The pair-join tests were conducted to examine the spatial pattern of individuals across the landscape, particularly to see whether we found patterns suggesting that individuals established significantly closer or further away from each other than would be expected at random. When analyzing all individuals of the same species within a population we found significant clustering of trees out to at least 100m in all populations (Fig S7a). This clustering persisted out to 600m at PP_COTTON_ and for PF_CASTLE_, though not for PP_CASTLE_*,* which showed clustering only to ~200m*.* At Anchor, the clustering rapidly dropped off over ~200-500m for PP_ANCHOR_ and ~400-600m for PF_ANCHOR_*.* We found similar but stronger associations of recruits with mature trees of the same species at all populations, an effect that grew stronger as more trees established, though with rapid drop offs after about 100m at all populations (Figure S7b). We found the same clustering for each of the three time periods we analyzed, with very little difference in the strength or pattern of associations through time (Figure S8).

*Mature trees and recruits between spp.* Associations of recruits to mature trees of a different species showed much weaker effects at large scales (Figure S7c). At Castle, some dis-association of mature *P. ponderosa* on recruits of *P. flexilis* exists from 100-200m. At smaller scales, we found strong association of mature trees of on species on recruits of the other at both sites (Figure S7c), which persisted out to ~50m. Interestingly, clustering was strong for recruits of *P. flexilis* around mature *P. ponderosa* from 0 – 50 m, while *P. ponderosa* recruits were only associated with *P. flexilis* adults beyond 15m. Note that panel C is only shown out to 50*m* because at the two sites where both species are present, there is insufficient numbers of recruits overlapping with mature adults of the opposite species at long-range distances to accurately calculate *g(r)* values. We note that this analysis was only done for Castle and Anchor populations, where both species were censused, and not at Cotton or Grass, where only *P. ponderosa* individuals were censused.

**Effective area analyses - a more spatially explicit analysis of dynamics**

*Assessing spatial associations of adults and recruits.* Density effects for sessile species are necessarily reflected in spatial associations, either negative or positive, and thus before modeling the temporal dynamics of our populations, we constructed a measure of habitat area available for new recruits, weighted by the relative likelihood of establishment. Within all six populations, recruitment is much more likely close to than far from existing adult trees (Figure 1). The distribution of establishment distances is relatively stable over time (Figure S2) within each population. These distances are non-random, as reflected in pair-join analyses (SI methods and SI7-8), and show both clustering around adults as well as potential competitive interactions, as indicated by relatively low numbers of recruits extremely close to adults (Figure S7, Figure 1). To include these effects, we parsed each habitat area into 0.5m x 0.5m grid points and calculated the distance from all available (unoccupied) grid points to the nearest mature tree; this was done separately for each decade and for each species, where both were present. We calculated the empirical density of all of these grid distances (*grid distances)* using the *density* function in R for each decade. This approximates the landscape density distribution of distance to nearest mature tree. We next calculated a density function for all observed establishment distances (*establishment distances)*, the distances from the location where an individual established to the closest mature tree that was in the landscape at that time. We multiplied these two density functions at intervals of 0.01m, and then summed these products. This yields a measure of available habitat area weighted by the likelihood of establishment, and thus contains information about both total area available and number and distribution of adults. More adults mean more of the habitat is in closer proximity to a mature tree, so establishment distances are necessarily smaller. We refer to this measure Effective Area (EA). We hypothesized that EA would provide a better predictor of establishment numbers than would the simple number of mature trees. We also used the distribution of establishment distances to look for predictable shifts in across time, using quantile regressions to examine trends in both extreme and median distances.

Because nearly all top population models included EA rather than number of mature adults, we also re-ran some simulations to indirectly use EA. To do this, we used a gam function (R function gamlss in GAMLSS package; Rigby and Stasinopoulos 2005) to fit the relationship between mature trees and EA at each population separately (a more direct approach than direct simulation of establishment distances). We used the top population models that included EA to predict number of recruits at each time step, then re-ran the model with the updated, estimated EA based on the new number mature trees. We note that at two populations, PF_CASTLE_ and PP_GRASS_, we had to use all of the data from all populations to fit the gam function to predict EA in the final two decades, as stochastic predictions were consistently falling far beyond the input data and the gam function was poorly extrapolating to these outliers.

*Test of EA vs Mature Trees as predictors.* Because almost all our top models supported EA over mature trees as better density variables, we re-ran the stochastic simulations using the relationship between EA and mature trees at each site to predict # recruits as a function of our EA models (Figure S6). We did not include both mature trees and EA in any model, due to their high correlation (Figure S4, r=0.95). The median simulations were very similar to median simulations of mature tree models, though in most cases, were slightly worse at tracking actual population growth than were our models using mature adults, and in some cases (most notably PF_ANCHOR_) slightly better. It should be noted that top PF_ANCHOR_ models without EA didn’t include mature trees.

**Effective Area (EA) Results**

Models that included our measure of EA were supported over models that used mature trees alone as a measure of density. Our initial calculation of EA relied on the observed spatial pattern of recruits and mature adults through time, and though the use of the gam function allowed for near perfect fitting of the relationship between # mature adults and EA for use in our simulations (Figure S4), our simulations predicted numbers of recruits through time and contained no information on locations of those hypothetical recruits. Thus, we suspect that spatial locations of individuals (not just number) play an important role in establishment rates, given that EA rather than mature adults was supported in top models, though simulation performance was not substantially improved by using EA vs. number of adults.

In our models that incorporated our measure of effective area (and its square) as a density effect rather than number of mature trees, all top population and species-wide models included the linear term and most included its square. Even at PF_ANCHOR,_ where the number of mature trees was not supported in the top models, our EA measure explicitly incorporating spatial data was strongly supported. The support for climate variables in EA models was strikingly similar to the models using # of mature trees (Tables S10 – S17). The squared EA term showed more consistent support in all top models than squared # of mature trees and was present in all top species-wide models.

Simulations using effective area rather than # of mature trees as predictors performed almost equally well (Figure S6), closely tracking actual population numbers. Simulations using EA at PF_ANCHOR_ performed far better than simulations using # of mature trees, showing that explicit inclusion of the spatial arrangement of recruits vastly improves predictive power there.

**JAGS Models without Negative Density Dependence**

To construct a biologically reasonable form of the negative binomial model that does not include any density dependence, but that does include the other explanatory factors supported in the best species models, we did analyses using an MCMC approach. We took this approach because the correct constraints to make a non-density-dependent model that includes other factors are difficult or impossible to accomplish using more standard glmm analysis packages. We fit the negative binomial model for recruit number using a log link function, and constraining the coefficient of ln (mature trees) in the linear function to be ~ 1. Because all of the explanatory variables are exponentiated when using a log link function, when the coefficient of ln(mature trees) is equal to one, the ratio of new recruits to existing adult trees is constant, regardless of density, corresponding to no negative or positive density dependence.

This framework allowed us, then, to compare models without density dependence to models with fitted density effects. We also ran JAGS models that allowed density dependence and found that these returned nearly identical coefficient estimates to the equivalent models run in *glm.nb.*

Our model takes the form:

$recruits= a+b\ln\left( M \right)+cM$ ,

where M is the # of mature adults, and *a, b,* and *c* are fitted coefficients. With a log-link function, the mean number of predicted recruits becomes

$$e^{a+b\ln\left( M \right)+cM}$$

$$= e^{a}M^{b}e^{cM}$$

This formulation allows for considerable flexibility in modeling density dependent effects. If b ~ 1, the predicted number of recruits is a constant function of the number of mature trees, indicating no density dependence. If b is less than 1, then the number of recruits increases more slowly, relative to the number of mature trees, indicating negative density dependence. Conversely, b > 1 indicates positive density dependence. The third term in the model ($e^{cM}$) adds more flexible functions for density dependence, such that with increasing number of big trees, the model can predict declining numbers of recruits (not just slower increases). In this case, the model is set up to be equivalent to a Ricker model, which is classically written as

$N_{t+1}={rN}_{t}*exp(-bN_{t})$

where $N_{t}$ is the number of individuals at time *t* (the same as *M* in our model, if we model non-overlapping generations such that $N_{t+1}$ is the number of new recruits at the next time step). *r* is the intrinsic growth rate, and *b* is a coefficient that typically adds a carrying capacity (*k*).

For each JAGS model we used 3 chains, 200 burn-in iterations, a thinning parameter of 3, and took 10,000 samples. To constrain the density coefficient, we set the used a normally distributed prior with a mean of 1 and a precision of 10^6^ for the log(BT) coefficient. All other priors were normally distributed with a mean of 0 and precision of 10^-6^.

**SUPPLEMENTAL FIGURES**

**
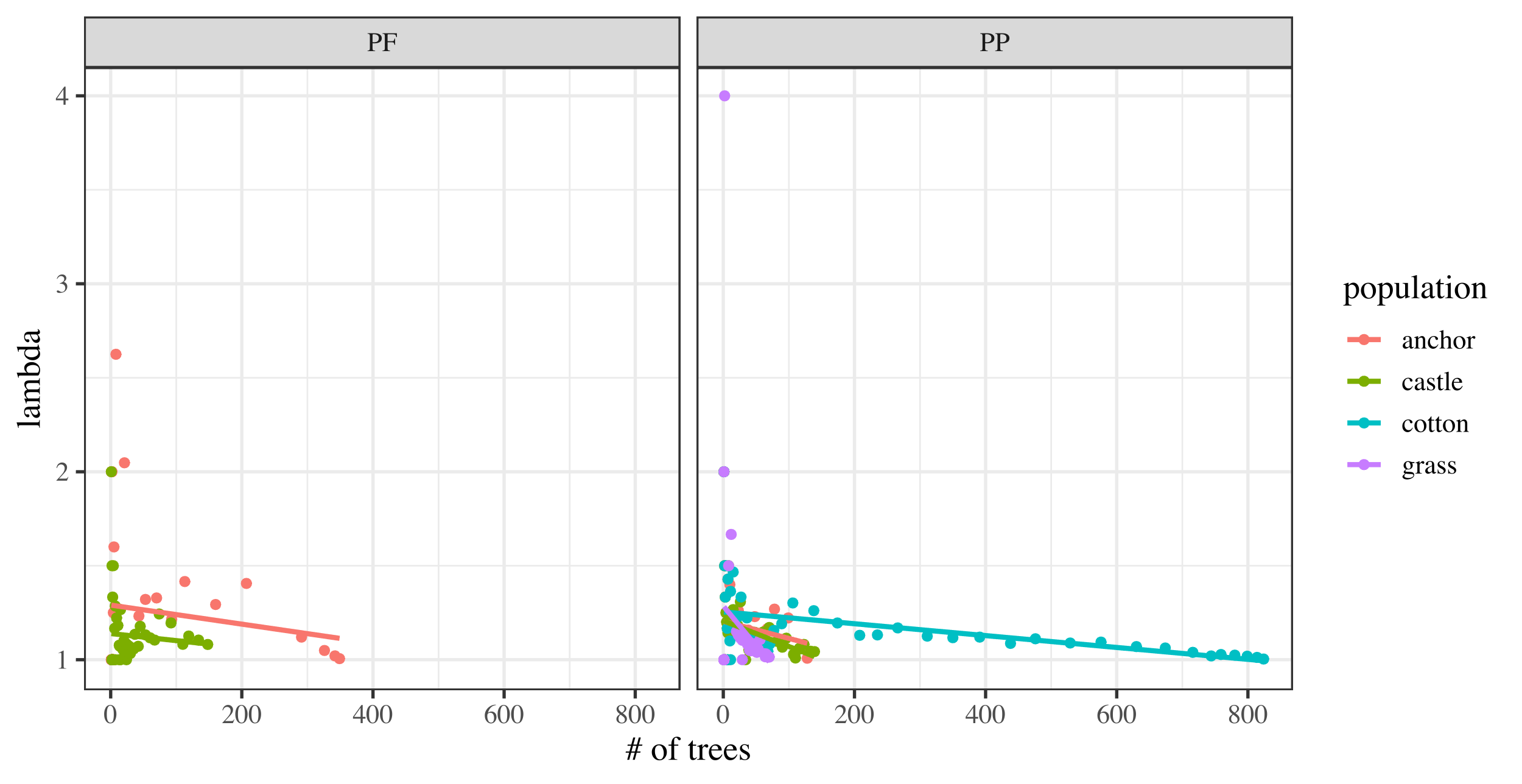
**

**Figure S1.** Decadal lambda values vs. density (# of trees) for *P. flexilis* and *P. ponderosa* populations, respectively. Lines show separate regressions for each population; regressions for PP_COTTON_ and PP_CASTLE_ are significant (p < 0.05).


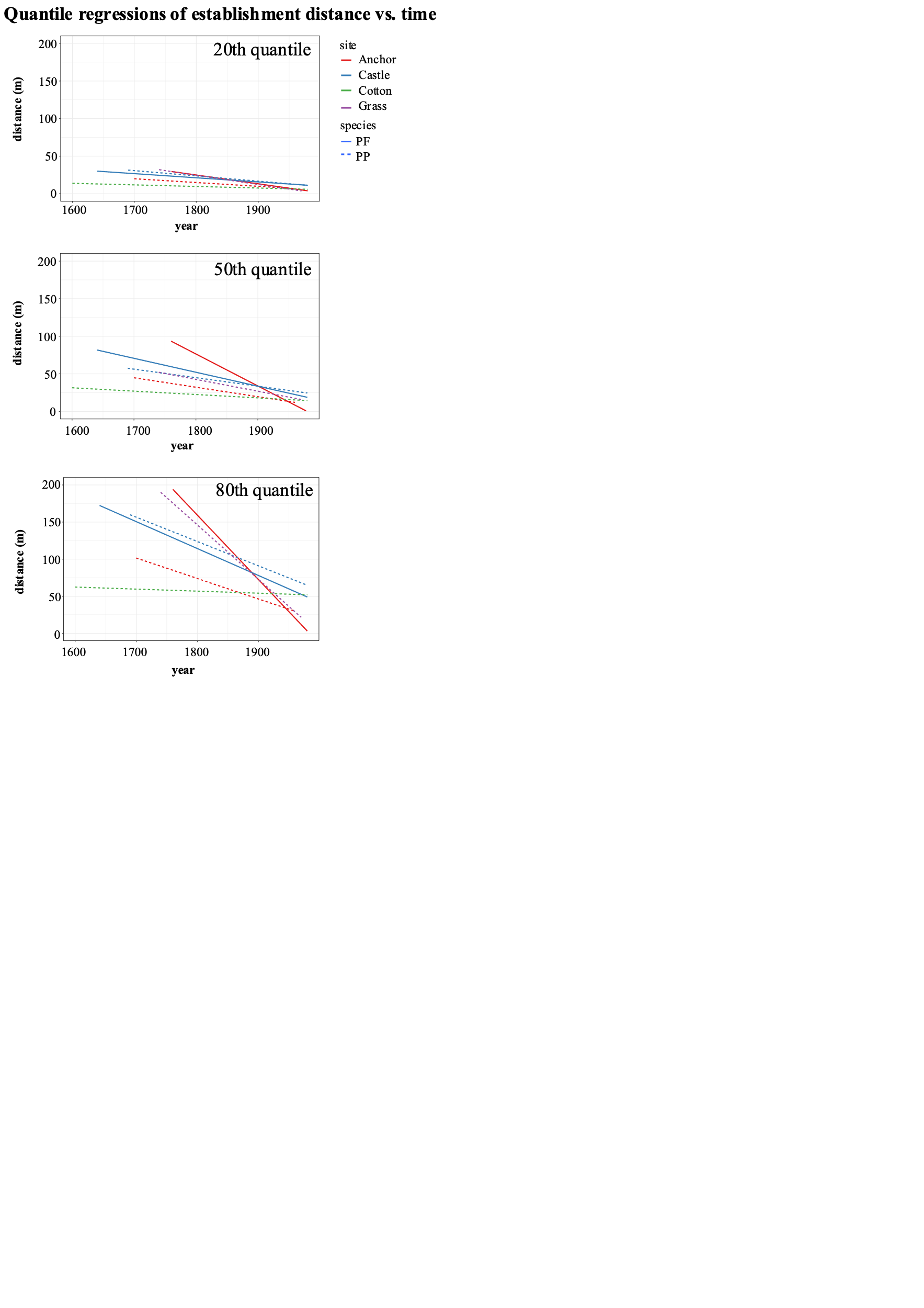


**Figure S2**. 20^th^, 50^th^, and 80^th^ quantile regressions of distance from establishment to the closest mature tree through time. Dotted lines indicate *P. ponderosa*, solid lines indicate *P. flexilis*, and colors indicate different populations. P-values for regressions of 20^th^ quantile values range from 10^-3^ – 0.14, for 50^th^ quantile range from 10^-3^ – 0.14, and for 80^th^ quantile range from 10^-4^ to 0.017.

**Figure S3.**

a. Median predictions of all classes of stochastic species-wide models shown; actual data shown in black; median of 500 simulations using the best species-wide model shown in red, using the top model without climate variables in blue, top model without density shown in orange, top model without site shown in pink, and non-stochastic predictions in yellow.

b. Annual proportional deviations (model predictions/observed) for each species-wide model type.

**
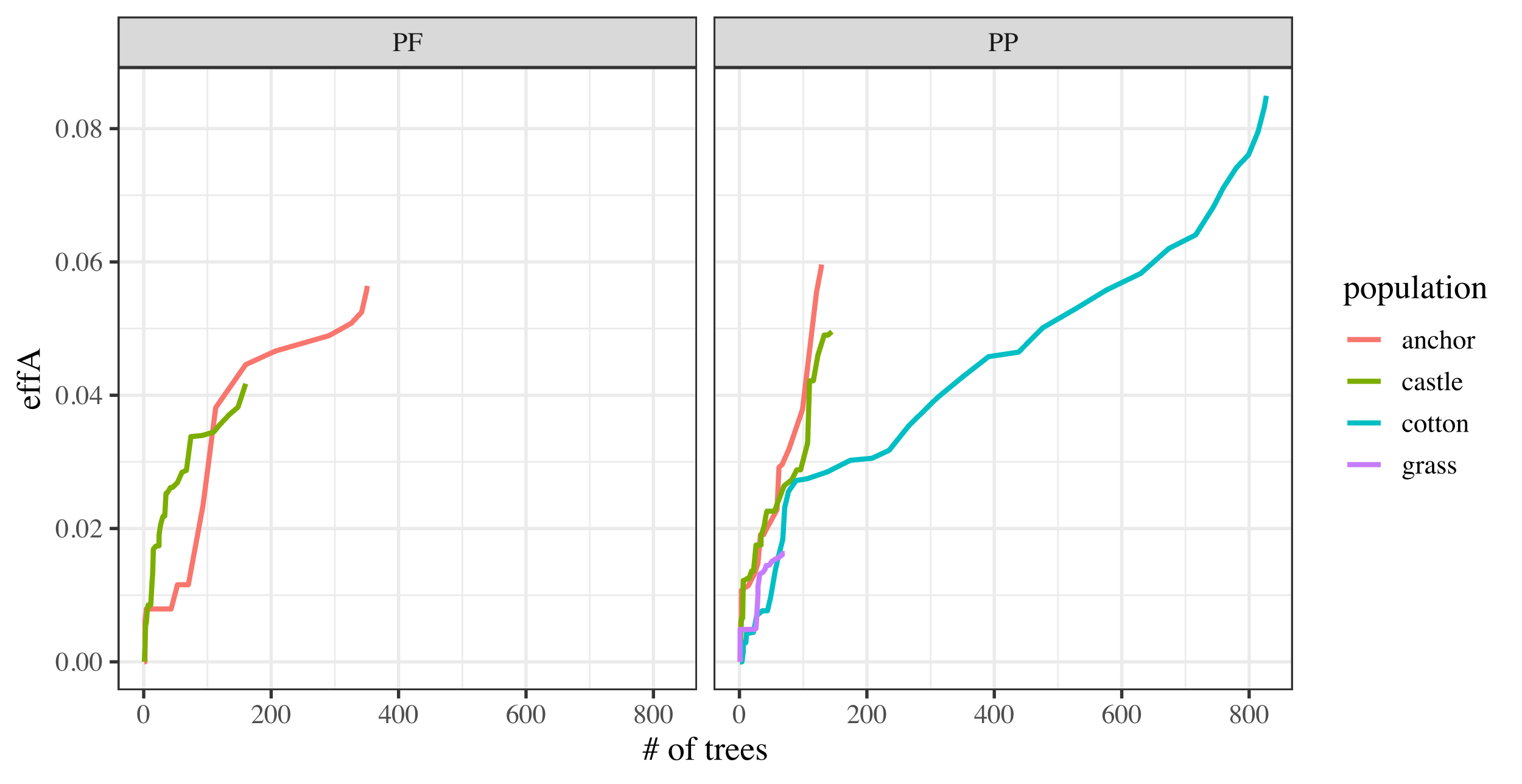
**

**Figure S4.** Effective area (EA) versus number of mature trees shown for each population *of P. flexilis* (A) and *P. ponderosa* (B). See text for calculation of effective area – a measure of available habitat area weighted by ‘suitability’, measured by proximity to mature trees.

**Figure S5.** Results of simulations using top population models of growth initiated during the decade when at least five adults were already present, and including any juveniles present during that decade, shown in grey. The black line indicates actual population numbers through time, and the red is the median of 500 simulations.

**Figure S6.** Results of 500 stochastic simulations using best population models that include effective area to predict population growth through time in grey. Gam function was used to estimate # reproductive adults as a function of effective area at each time step using the existing relationship between EA and mature trees at each population. See methods for details. Median of 500 simulations shown in red, actual population numbers in black.

**
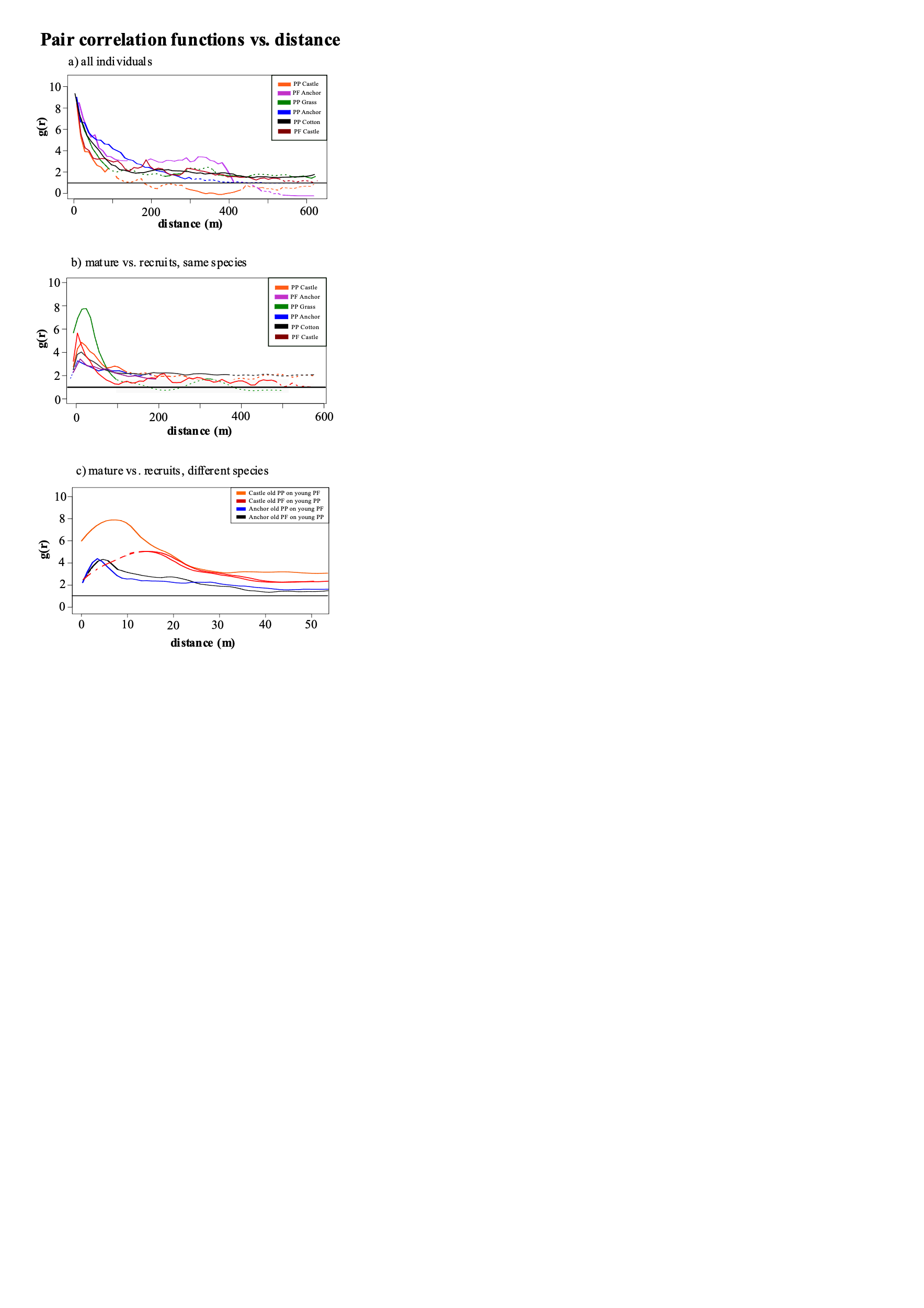
**

**Figure S7.** Pair correlation functions for: (a) all individuals within each population; (b) mature vs. new recruits of the same species; and (c) recruits around adults of the other species. G(r) values >1 indicate clustering, values <1 indicate disassociation from a random Poisson point process and are plotted against distance (m). Dotted sections indicate areas within 90% confidence limits, while solid sections indicate significant clustering or disassociation. See SI methods for confidence interval construction.

**Figure S8.** a) Pair correlations for time period when populations had 10 mature trees (but including juveniles in the calculation), & b) for time period when population size was closest to half of ending population size (again including juveniles). Present day PCF functions are displayed in S7a.

**Figure S9.** Map of site locations within Bighorn Basin, Wyoming. Note that co-occuring *Pinus flexilis* and P. ponderosa were censused at two of our sites (Anchor and Castle Garden). Points show occurrence data from GBIF (which include iNaturalist and eBird data) of all *P. ponderosa* and *P. flexilis* within the Basin. Documented sightings of Clark’s nutcracker (*Nucifraga columbiana*, n = 15), known to be a long-distance disperser of seeds, are also shown.

**
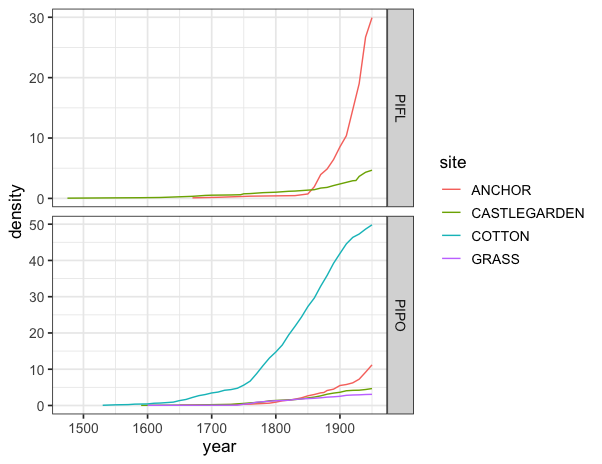
**

**Figure S10**: Densities of each population (number of individuals per km^2^ of delineated habitat area). Densities are calculated including only trees from same species and site.

**
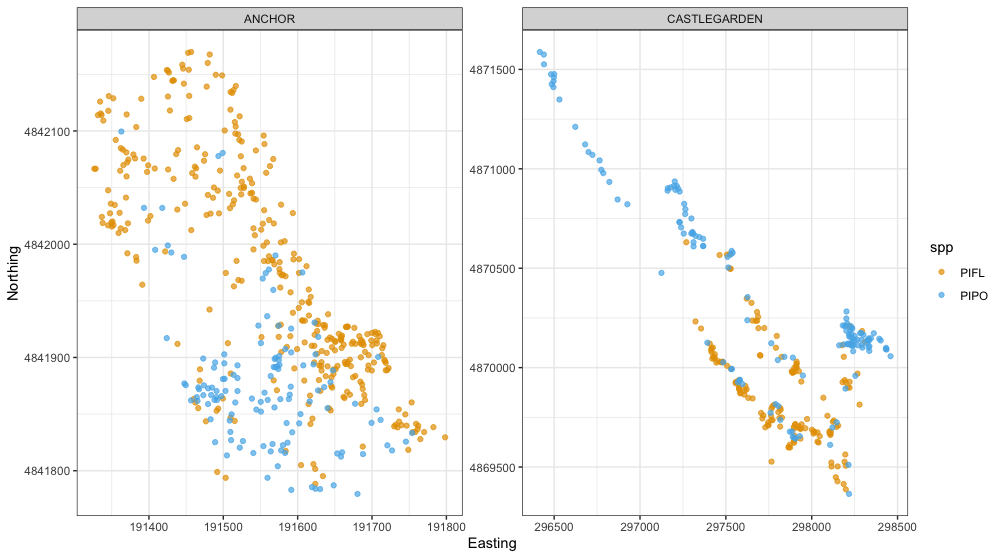
**

**Figure S11:** Distributions of *P. ponderosa* and *P. flexilis* at two sites where they co-occur. While local separation is apparent, the spatial segregation does not correspond to obvious aspect, elevational, or soil type differences.

SUPPLEMENTAL TABLES

**Table S1. Location and size of each population.**

| **Population** | **Latitude** | **Longitude** | **Elevation(m)** | **Year of first establishment event** | **Available habitat size*** | **# of trees (as of 1950)** |
| --- | --- | --- | --- | --- | --- | --- |
| Castle Garden, P. ponderosa | 43.96 N | 107.52 W | 1450 - 1550 | 1592 | 26.34 km^2^ | 123 |
| Castle Garden, P. flexilis | 43.96 N | 107.52 W | 1450 - 1550 | 1470 | 26.34 km^2^ | 119 |
| Anchor Dam, P. ponderosa | 43.67 N | 108.83 W | 1950 - 2050 | 1655 | 10.89 km^2^ | 121 |
| Anchor Dam, P. flexilis | 43.67 N | 108.83 W | 1950 - 2050 | 1670 | 10.89 km^2^ | 326 |
| Grass Creek, P. ponderosa | 43.88 N | 108.63 W | 1700 - 1900 | 1605 | 22.11 km^2^ | 68 |
| Cottonwood Creek, P. ponderosa | 43.80 N | 108.70 W | 1700 - 1850 | 1531 | 16.84 km^2^ | 799 |

*****see methods in main text for estimation of ‘available habitat’ delineation in each location

**Table S2. Correlations in two measures of growth.**

**Upper triangle: Pearson correlation coefficients of #recruits/#big trees through time between populations. PP indicates *Pinus ponderosa,* and PF indicates *Pinus flexilis,* which is followed by site name. Lower triangle, in red: Pearson correlation coefficients of lambda through time between populations. PP indicates *Pinus ponderosa,* and PF indicates *Pinus flexilis,* which is followed by site name.**

|  | PF_CASTLE_ | PP_CASTLE_ | PF_ANCHOR_ | PP_ANCHOR_ | PP_GRASS_ | PP_COTTON_ |
| --- | --- | --- | --- | --- | --- | --- |
| PF_CASTLE_ |  | 0.112 | 0.079 | -0.241 | -0.049 | 0.349 |
| PP_CASTLE_ | 0.263 |  | -0.192 | 0.229 | 0.479 | 0.575 |
| PF_ANCHOR_ | -0.053 | -0.093 |  | -0.168 | -0.179 | -0.299 |
| PP_ANCHOR_ | -0.240 | 0.072 | -0.038 |  | 0.010 | 0.423 |
| PP_GRASS_ | -0.012 | 0.299 | -0.123 | 0.149 |  | 0.267 |
| PP_COTTON_ | -0.155 | 0.075 | -0.190 | 0.030 | 0.111 |  |

**Top population models.**

**Table S3.** Top models of establishment at PP_CASTLE_. All top models that in sum include 90% AICc weights shown. MT= # mature trees, log(MT) = natural logarithm of # of mature trees. Climate variables abbreviated to indicate decade followed by variable type, e.g. 1P = one decade post-establishment precipitation average, 2T=two decade temperature average post-establishment, etc. Top (and only possible) 3 models without climate variables are shaded in blue.

| **Intercept** | **MT** | **Log(MT)** | **1P** | **1T** | **2P** | **2T** | **3P** | **3T** | **Df** | **logLik** | **AICc** | **Delta** | **Weight** |
| --- | --- | --- | --- | --- | --- | --- | --- | --- | --- | --- | --- | --- | --- |
| - |  | + |  |  |  | - |  |  | 4 | -69.271079 | 146.542157 | 0 | 0.05461538 |
| - | - | + |  |  |  | - |  |  | 5 | -68.688275 | 147.376549 | 0.83439219 | 0.03598562 |
| - |  | + |  | - |  | - |  |  | 5 | -68.843362 | 147.686724 | 1.14456731 | 0.03081593 |
| - |  | + |  |  |  | - | + |  | 5 | -69.004959 | 148.009917 | 1.46776021 | 0.02621772 |
| - | - | + |  |  |  |  |  |  | 4 | -71.54737 | 151.09474 | 4.55258258 | 0.00560705 |
| - |  | + |  |  |  |  |  |  | 3 | -72.602906 | 151.205812 | 4.66365455 | 0.00530414 |
| + |  |  |  |  |  |  |  |  | 2 | -88.923917 | 181.847834 | 35.3056771 | 1.18E-09 |

**Table S4.** Top models of establishment at PP_ANCHOR_. All top models that in sum include at least 90% AICc weights shown. MT= # mature trees, log(MT) = natural logarithm of # of mature trees. Climate variables abbreviated to indicate decade followed by variable type, e.g. 1P = one decade post-establishment precipitation average, 2T=two decade temperature average post-establishment, etc. Top (and only possible) 3 models without climate variables are shaded in blue.

| **Intercept** | **MT** | **Log(MT)** | **1P** | **1T** | **2P** | **2T** | **3P** | **3T** | **Df** | **logLik** | **AICc** | **Delta** | **Weight** |
| --- | --- | --- | --- | --- | --- | --- | --- | --- | --- | --- | --- | --- | --- |
| - |  | + |  | + | - |  | + | - | 7 | -46.524391 | 107.048782 | 0 | 0.12379917 |
| - |  | + |  | + |  |  | + | - | 6 | -47.962706 | 107.925412 | 0.8766297 | 0.07986564 |
| - |  | + |  | + | - |  |  | - | 6 | -48.248898 | 108.497796 | 1.4490140 | 0.05998854 |
| - |  | + | - | + | - |  | + | - | 8 | -46.415184 | 108.830367 | 1.7815850 | 0.05079857 |
| - |  | + |  | + | - | + | + | - | 8 | -46.498724 | 108.997449 | 1.9486667 | 0.04672724 |
| - | - | + |  | + | - |  | + | - | 8 | -46.515151 | 109.030302 | 1.9815198 | 0.04596594 |
| - |  | + |  |  |  |  |  |  | 3 | -54.845827 | 115.691654 | 8.6428716 | 0.00164415 |
| - | + | + |  |  |  |  |  |  | 4 | -54.842508 | 117.685017 | 10.636234 | 0.00060686 |
| + |  |  |  |  |  |  |  |  | 2 | -79.215509 | 162.431019 | 55.382236 | 1.17E-13 |

**Table S5.** Top models of establishment at PP_COTTON_. All top models that in sum include at least 90% AICc weights shown. MT= # mature trees, log(MT) = natural logarithm of # of mature trees. Climate variables abbreviated to indicate decade followed by variable type, e.g. 1P = one decade post-establishment precipitation average, 2T=two decade temperature post-establishment average, etc. Top (and only possible) 3 models without climate variables are shaded in blue. Note that the top overall model is also the top model without climate effects, but row is repeated for both categories for clarity.

| **Intercept** | **MT** | **Log(MT)** | **1P** | **1T** | **2P** | **2T** | **3P** | **3T** | **Df** | **logLik** | **AICc** | **Delta** | **Weight** |
| --- | --- | --- | --- | --- | --- | --- | --- | --- | --- | --- | --- | --- | --- |
| **-** | **-** | **+** |  |  |  |  |  |  | 4 | -131.36509 | 270.730178 | 0 | 0.0491 |
| **-** | **-** | **+** |  | **-** |  |  |  |  | 5 | -130.81057 | 271.621148 | 0.89097 | 0.0314 |
| **-** | **-** | **+** |  |  |  |  |  | **-** | 5 | -130.89736 | 271.794719 | 1.0645408 | 0.0288 |
| **-** | **-** | **+** |  |  |  | **-** |  |  | 5 | -131.09315 | 272.186308 | 1.4561295 | 0.0237 |
| **-** | **-** | **+** | **+** |  |  |  |  |  | 5 | -131.23301 | 272.466028 | 1.7358499 | 0.0206 |
| **-** | **-** | **+** |  |  |  |  |  |  | 4 | -131.36509 | 270.730178 | 0 | 0.02454789 |
| **+** |  | **+** |  |  |  |  |  |  | 3 | -138.56749 | 283.134978 | 12.404798 | 4.97E-05 |
| **+** |  |  |  |  |  |  |  |  | 2 | -167.44134 | 338.882678 | 68.152499 | 3.90E-17 |

**Table S6.** Top models of establishment at PP_GRASS_. All top models that in sum include at least 90% AICc weights shown. All top models with AICc weight > 0.01 shown. MT= # mature trees, log(MT) = natural logarithm of # of mature trees. Climate variables abbreviated to indicate decade followed by variable type, e.g. 1P = one decade post-establishment precipitation average, 2T=two decade temperature post-establishment average, etc. Top (and only possible) 3 models without climate variables are shaded in blue. Note that one of the top models l is also a top model without climate effects, but row is repeated for both categories for clarity.

| **Intercept** | **MT** | **Log(MT)** | **1P** | **1T** | **2P** | **2T** | **3P** | **3T** | **Df** | **logLik** | **AICc** | **Delta** | **Weight** |
| --- | --- | --- | --- | --- | --- | --- | --- | --- | --- | --- | --- | --- | --- |
| **+** | **-** | **+** |  |  |  |  |  | **+** | 5 | -62.44697 | 134.893939 | 0 | 0.04131257 |
| **+** | **-** | **+** |  |  |  |  |  |  | 4 | -63.870159 | 135.740318 | 0.8463793 | 0.02705785 |
| **+** | **-** | **+** | **+** |  |  |  |  |  | 5 | -62.95142 | 135.902839 | 1.0089002 | 0.02494608 |
| **+** | **-** | **+** | **+** |  |  |  |  | **+** | 6 | -62.060465 | 136.12093 | 1.2269913 | 0.02236889 |
| **+** | **-** | **+** |  |  | **+** |  |  | **+** | 6 | -62.140047 | 136.280094 | 1.3861552 | 0.02065772 |
| **+** | **-** | **+** |  |  |  |  |  |  | 4 | -63.870159 | 135.740318 | 0.8463793 | 0.02705785 |
| **+** |  | **+** |  |  |  |  |  |  | 3 | -65.278754 | 136.557507 | 1.6635680 | 0.01798221 |
| **+** |  |  |  |  |  |  |  |  | 2 | -67.42925 | 138.858501 | 3.9645616 | 0.005691 |

**Table S7.** Top models of establishment at PF_CASTLE_. All top models that in sum include at least 90% AICc weights shown. MT= # mature trees, log(MT) = natural logarithm of # of mature trees. Climate variables abbreviated to indicate decade followed by variable type, e.g. 1P = one decade post-establishment precipitation average, 2T=two decade temperature average post-establishment, etc. Top (and only possible) 3 models without climate variables are shaded in blue. Note that one of the top models is also a top model without climate effects, but row is repeated for both categories for clarity.

| **Intercept** | **MT** | **Log(MT)** | **1P** | **1T** | **2P** | **2T** | **3P** | **3T** | **Df** | **logLik** | **AICc** | **Delta** | **Weight** |
| --- | --- | --- | --- | --- | --- | --- | --- | --- | --- | --- | --- | --- | --- |
| **-** | **+** | **+** |  |  | **+** |  |  |  | 5 | -70.833731 | 151.667462 | 0 | 0.0278261 |
| **-** | **+** | **+** |  |  |  |  |  |  | 4 | -71.847021 | 151.694042 | 0.0265798 | 0.0274978 |
| **-** | **+** | **+** |  | **-** | **+** |  |  |  | 6 | -70.499021 | 152.998042 | 1.3305797 | 0.0268695 |
| **-** | **+** | **+** |  | **-** |  |  |  |  | 5 | -71.510888 | 153.021775 | 1.3543133 | 0.0263527 |
| **-** | **+** | **+** |  |  |  |  |  | **-** | 5 | -71.534003 | 153.068005 | 1.4005435 | 0.0207737 |
| **-** | **+** | **+** |  |  | **+** |  |  | **-** | 6 | -70.553423 | 153.106846 | 1.4393844 | 0.0205896 |
| **-** | **+** | **+** |  |  |  |  |  |  | 4 | -71.847021 | 151.694042 | 0.0265798 | 0.0274978 |
| **-** |  | **+** |  |  |  |  |  |  | 3 | -77.852695 | 161.705389 | 15.744618 | 3.58E-04 |
| **+** |  |  |  |  |  |  |  |  | 2 | -98.739718 | 201.479435 | 55.518664 | 8.26E-12 |

**Table S8.** Top models of establishment at PF_ANCHOR_. All top models that in sum include at least 90% AICc weights shown. MT= # mature trees, log(MT) = natural logarithm of # of mature trees. Climate variables abbreviated to indicate decade followed by variable type, e.g. 1P = one decade post-establishment precipitation average, 2T=two decade temperature average post-establishment, etc. Top (and only possible) 3 models without climate variables are shaded in blue.

| **Intercept** | **MT** | **Log(MT)** | **1P** | **1T** | **2P** | **2T** | **3P** | **3T** | **Df** | **logLik** | **AICc** | **Delta** | **Weight** |
| --- | --- | --- | --- | --- | --- | --- | --- | --- | --- | --- | --- | --- | --- |
| **-** |  | **+** | **+** | **-** |  |  |  | **-** | 6 | -66.943486 | 145.886973 | 0 | 0.02726848 |
| **-** |  | **+** |  | **-** |  |  |  | **-** | 5 | -68.191083 | 146.382166 | 0.4951929 | 0.02128782 |
| **-** |  | **+** | **+** | **-** |  |  | **-** | **-** | 7 | -66.353479 | 146.706958 | 0.8199854 | 0.01809686 |
| **-** |  | **+** | **+** | **-** |  | **-** |  | **-** | 7 | -66.617334 | 147.234669 | 1.3476962 | 0.01389992 |
| **-** |  | **+** |  | **-** |  | **-** |  | **-** | 6 | -67.694975 | 147.389951 | 1.5029780 | 0.01286155 |
| **-** |  | **+** | **+** | **-** | **+** |  |  | **-** | 7 | -66.794936 | 147.589872 | 1.7028992 | 0.01163807 |
| **+** |  | **+** |  |  |  |  |  |  | 3 | -75.468594 | 156.937187 | 15.149764 | 2.72E-05 |
| **+** | **-** | **+** |  |  |  |  |  |  | 4 | -75.221363 | 158.442725 | 16.655302 | 1.28E-05 |
| **+** |  |  |  |  |  |  |  |  | 2 | -84.798814 | 173.597629 | 31.810205 | 6.55E-09 |

**Species-wide mixed models.**

**Table S9.** Top species-wide models of establishment for all *P. ponderosa* populations, with population (S) as a fixed effect. Climate variables abbreviated to indicate decade followed by variable type, e.g. 1P = one decade post-establishment precipitation average, 2T=two decade temperature average post-establishment, etc. Here we refer to population as site (S), so as not to confuse with precip (P) variables. Top models without climate variables are shown in blue, and top models without site in orange. * indicates that the term was included (if a fixed effect), and + / - indicates the sign of the effect. Note that the top overall model is also the top model without climate effects, but row is repeated for both categories for clarity.

| **Intercept** | **S** | **MT** | **Log(MT)** | **1P** | **1T** | **2P** | **2T** | **3P** | **3T** | **MT*S** | **Log(MT)*S** | **1P*S** | **1T*S** | **2P*S** | **2T*S** | **3P*S** | **3T*S** | **df** | **logLik** | **AICc** | **delta** | **weight** |
| --- | --- | --- | --- | --- | --- | --- | --- | --- | --- | --- | --- | --- | --- | --- | --- | --- | --- | --- | --- | --- | --- | --- |
| - | * | + | + |  |  |  |  |  |  | * |  |  |  |  |  |  |  | 10 | -327.35713 | 674.7142 | 0.0000 | 0.0375 |
| - | * | + | + |  |  |  | - |  |  | * |  |  |  |  |  |  |  | 11 | -326.8122 | 675.6244 | 0.9101 | 0.0238 |
| - | * | + | + |  |  |  |  |  | - | * |  |  |  |  |  |  | * | 14 | -323.88231 | 675.7646 | 1.0504 | 0.0222 |
| - | * | + | + | + |  |  |  |  |  | * |  |  |  |  |  |  |  | 11 | -327.20326 | 676.4065 | 1.6923 | 0.0161 |
| - | * | + | + |  |  |  |  | + |  | * |  |  |  |  |  |  |  | 11 | -327.23968 | 676.4793 | 1.7651 | 0.0155 |
| - | * | + | + |  |  |  |  |  | - | * |  |  |  |  |  |  |  | 11 | -327.26909 | 676.5381 | 1.8239 | 0.0151 |
| - | * | + | + |  |  |  |  |  |  | * |  |  |  |  |  |  |  | 10 | -327.35713 | 674.7142 | 0.0000 | 0.0375 |
| - | * | - | + |  |  |  |  |  |  |  | * |  |  |  |  |  |  | 10 | -329.23045 | 678.4609 | 3.74664 | 0.00576 |
| - | * | + | + |  |  |  |  |  |  | * | * |  |  |  |  |  |  | 13 | -326.53957 | 679.0791 | 4.36488 | 0.00423 |
| - | * |  | + |  |  |  |  |  |  |  | * |  |  |  |  |  |  | 9 | -337.48284 | 692.9656 | 18.2514 | 4.0813E-06 |
| + |  |  | + |  |  |  |  |  |  |  |  |  |  |  |  |  |  | 3 | -363.10221 | 732.2044 | 57.4901 | 1.2313E-14 |
| - |  |  | + |  |  |  |  |  | - |  |  |  |  |  |  |  |  | 4 | -362.91546 | 733.8309 | 59.1166 | 5.4625E-15 |
| - |  |  | + |  |  |  |  | + |  |  |  |  |  |  |  |  |  | 4 | -362.99313 | 733.9862 | 59.2719 | 5.05E-15 |

**Table S10.** Top species-wide models of establishment for all *P. flexilis* populations, with population (S) as a fixed effect. Climate variables abbreviated to indicate decade followed by variable type, e.g. 1P = one decade post-establishment precipitation average, 2T=two decade temperature average post-establishment, etc. Here we refer to population as site (S), so as not to confuse with precip (P) variables. Top models without climate variables are shown in blue, and top models without site in orange. * indicates that the term was included (if a fixed effect), and + / - indicates the sign of the effect.

| **Intercept** | **S** | **MT** | **Log(MT)** | **1P** | **1T** | **2P** | **2T** | **3P** | **3T** | **MT*S** | **Log(MT)*S** | **1P*S** | **1T*S** | **2P*S** | **2T*S** | **3P*S** | **3T*S** | **df** | **logLik** | **AICc** | **delta** | **weight** |
| --- | --- | --- | --- | --- | --- | --- | --- | --- | --- | --- | --- | --- | --- | --- | --- | --- | --- | --- | --- | --- | --- | --- |
| **-** | ***** | **-** | **+** |  | **-** |  |  |  | **-** | ***** | ***** |  |  |  |  |  | ***** | 10 | -142.34122 | 304.682446 | 0 | 0.03062 |
| **-** | ***** | **-** | **+** |  | **-** |  |  |  | **-** | ***** | ***** |  | ***** |  |  |  | ***** | 11 | -141.51407 | 305.028145 | 0.3456992 | 0.02583 |
| **-** | ***** | **-** | **+** | **+** | **-** |  |  |  | **-** | ***** | ***** | ***** |  |  |  |  | ***** | 12 | -140.53226 | 305.064516 | 0.38207 | 0.02537 |
| **-** | ***** | **-** | **+** | **+** | **-** |  |  |  | **-** | ***** | ***** | ***** | ***** |  |  |  | ***** | 13 | -139.67478 | 305.349565 | 0.6671191 | 0.02201 |
| **-** | ***** | **-** | **+** | **+** | **-** |  |  |  | **-** | ***** | ***** |  |  |  |  |  | ***** | 11 | -141.81097 | 305.621949 | 0.939503 | 0.01919 |
| **-** | ***** | **-** | **+** | **+** | **-** |  |  |  | **-** | ***** | ***** |  | ***** |  |  |  | ***** | 12 | -140.81801 | 305.636027 | 0.953581 | 0.01906 |
| **-** |  | **+** | **+** |  | **-** |  |  |  | **-** |  |  |  |  |  |  |  |  | 6 | -166.95758 | 345.915155 | 41.2327093 | 3.4179E-11 |
| **-** |  | **+** | **+** |  | **-** |  | **-** |  | **-** |  |  |  |  |  |  |  |  | 7 | -166.43093 | 346.861852 | 42.1794063 | 2.13E-11 |
| **-** |  | **+** | **+** | **+** | **-** |  |  |  | **-** |  |  |  |  |  |  |  |  | 7 | -166.60328 | 347.206562 | 42.5241161 | 1.7937E-11 |
| **-** | ***** | **-** | **+** |  |  |  |  |  | ***** |  | ***** |  |  |  |  |  |  | 7 | -154.24999 | 322.499974 | 17.8175284 | 4.1562E-06 |
| **+** | ***** |  | **+** |  |  |  |  |  |  |  |  |  |  |  |  |  |  | 4 | -160.32213 | 328.644252 | 23.9618064 | 1.925E-07 |
| **+** | ***** |  | **+** |  |  |  |  |  |  |  | ***** |  |  |  |  |  |  | 5 | -160.21903 | 330.438057 | 25.7556112 | 7.8475E-08 |

**Table S11.** Coefficient values for density terms in top population models. A coefficient > 1 for *ln*(MT) indicates positive density dependence, and a coefficient < 1 indicates negative density dependence. Mature trees (MT) is exponentiated with the log-link function, so a this term represents a more flexible, nonlinear density term.

| **Model** | **Ln(# Mature Trees) coefficient value** | **Mature trees (MT) coefficient value** |
| --- | --- | --- |
| PF Anchor | 1.488 | NA |
| PP Anchor | 0.748 | NA |
| PF Castle | 0.265 | 0.039 |
| PP Castle | 0.499 | NA |
| PP Cotton | 0.795 | -0.003 |
| PP Grass | 0.623 | -0.045 |

**Table S12.** Ghost tree effects. A ‘time’ variable [time since population initiation] was added to check for evidence of cryptic past mortality. The coefficient for time effect in the top model with ‘time’ is given below for each population model. If mature adults died in the past and are no longer detectable, then we’d expect that the # of recruits per censused adult tree should decrease as time since population initiation increases, and thus, the coefficient estimate should be (-).

Coefficient estimates for “time” variable.

| **Model** | **Time coefficient** |
| --- | --- |
| PF Anchor | 2.323 |
| PP Anchor | 1.572 |
| PF Castle | 3.240 |
| PP Castle | 2.583 |
| PP Cotton | 4.281 |
| PP Grass | 3.750 |

**EA population models.**

**Table S13.** Top models of establishment using effective area at PF_CASTLE_. All top models that in sum include at least 90% AICc weights shown. EA= effective area. Climate variables abbreviated to indicate decade followed by variable type, e.g. 1P = first decade precipitation, 2T=second decade temperature, etc.

| **Intercept** | **EA** | **Log(EA)** | **1P** | **1T** | **2P** | **2T** | **3P** | **3T** | **Df** | **logLik** | **AICc** | **Delta** | **Weight** |
| --- | --- | --- | --- | --- | --- | --- | --- | --- | --- | --- | --- | --- | --- |
| **-** | **+** | **-** |  |  | **+** |  |  |  | 5 | -67.980386 | 145.960771 | 0 | 0.09388 |
| **-** | **+** | **-** |  |  |  |  |  |  | 4 | -69.222444 | 146.444888 | 0.48411713 | 0.07369 |

**Table S14.** Top models of establishment using effective area at PF_ANCHOR_. All top models that in sum include at least 90% AICc weights shown. EA= effective area. Climate variables abbreviated to indicate decade followed by variable type, e.g. 1P = first decade precipitation, 2T=second decade temperature, etc.

| **Intercept** | **EA** | **Log(EA)** | **1P** | **1T** | **2P** | **2T** | **3P** | **3T** | **Df** | **logLik** | **AICc** | **Delta** | **Weight** |
| --- | --- | --- | --- | --- | --- | --- | --- | --- | --- | --- | --- | --- | --- |
| **+** | **+** | **+** |  | **-** |  | **-** |  | **-** | 7 | -64.123458 | 142.246917 | 0.45949376 | 0.04207 |
| **+** | **+** | **+** |  | **-** |  |  |  | **-** | 6 | -65.22702 | 142.45404 | 0.66661731 | 0.0379 |
| **+** | **+** | **+** | **+** | **-** |  | **-** |  | **-** | 8 | -63.594228 | 143.188456 | 1.40103327 | 0.02627 |
| **+** | **+** | **+** |  | **-** | **-** | **-** |  | **-** | 8 | -63.678965 | 143.357929 | 1.57050608 | 0.02414 |

**Table S15.** Top models of establishment using effective area at PP_CASTLE_. All top models that in sum include at least 90% AICc weights shown. EA= effective area. Climate variables abbreviated to indicate decade followed by variable type, e.g. 1P = first decade precipitation, 2T=second decade temperature, etc.

| **Intercept** | **EA** | **Log(EA)** | **1P** | **1T** | **2P** | **2T** | **3P** | **3T** | **Df** | **logLik** | **AICc** | **Delta** | **Weight** |
| --- | --- | --- | --- | --- | --- | --- | --- | --- | --- | --- | --- | --- | --- |
| **+** | **-** | **+** |  |  |  | **-** |  |  | 5 | -68.629547 | 147.259094 | 0.71693689 | 0.03816 |
| **+** | **-** | **+** | **-** |  |  | **-** |  |  | 6 | -68.291121 | 148.582242 | 2.04008457 | 0.0196 |
| **+** | **-** | **+** |  |  | **-** | **-** |  |  | 6 | -68.406565 | 148.813129 | 2.27097223 | 0.01754 |
| **+** | **-** | **+** |  | **-** |  | **-** |  |  | 6 | -68.442573 | 148.885146 | 2.34298866 | 0.0169 |
| **+** | **-** | **+** |  |  |  | **-** | **+** |  | 6 | -68.480743 | 148.961486 | 2.41932893 | 0.01629 |
| **+** | **-** | **+** |  |  |  | **-** |  | **-** | 6 | -68.629148 | 149.258297 | 2.71613941 | 0.01404 |

**Table S16.** Top models of establishment using effective area at PP_ANCHOR_. All top models that in sum include at least 90% AICc weights shown. EA= effective area. Climate variables abbreviated to indicate decade followed by variable type, e.g. 1P = first decade precipitation, 2T=second decade temperature, etc.

| **Intercept** | **EA** | **Log(EA)** | **1P** | **1T** | **2P** | **2T** | **3P** | **3T** | **Df** | **logLik** | **AICc** | **Delta** | **Weight** |
| --- | --- | --- | --- | --- | --- | --- | --- | --- | --- | --- | --- | --- | --- |
| **+** | **-** | **+** |  | **+** | **-** |  | **+** | **-** | 8 | -46.98772 | 109.975439 | 2.92665697 | 0.03865 |
| **+** | **-** | **+** |  | **+** |  |  | **+** | **-** | 7 | -48.183595 | 110.367191 | 3.31840843 | 0.02755 |
| **+** | **-** | **+** |  | **+** | **-** |  |  | **-** | 7 | -48.719913 | 111.439825 | 4.39104305 | 0.01377 |
| **+** | **-** | **+** | **-** | **+** | **-** |  | **+** | **-** | 9 | -46.890347 | 111.780695 | 4.73191231 | 0.01161 |
| **+** | **-** | **+** |  | **+** | **-** | **-** | **+** | **-** | 9 | -46.984062 | 111.968123 | 4.91934114 | 0.01058 |

**Table S17.** Top models of establishment using effective area at PP_COTTON_. All top models that in sum include at least 90% AICc weights shown. EA= effective area. Climate variables abbreviated to indicate decade followed by variable type, e.g. 1P = first decade precipitation, 2T=second decade temperature, etc.

| **Intercept** | **EA** | **Log(EA)** | **1P** | **1T** | **2P** | **2T** | **3P** | **3T** | **Df** | **logLik** | **AICc** | **Delta** | **Weight** |
| --- | --- | --- | --- | --- | --- | --- | --- | --- | --- | --- | --- | --- | --- |
| **+** | **-** | **+** |  |  |  |  |  | **-** | 5 | -129.62664 | 269.253284 | 0 | 0.05137095 |
| **+** | **-** | **+** | **+** |  |  |  |  | **-** | 6 | -128.809 | 269.617999 | 0.36471509 | 0.04280758 |
| **+** | **-** | **+** |  | **-** |  |  |  | **-** | 6 | -129.0142 | 270.028395 | 0.77511111 | 0.03486618 |
| **+** | **-** | **+** | **+** | **-** |  |  |  | **-** | 7 | -128.19277 | 270.38553 | 1.13224624 | 0.02916443 |

**Table S18.** Top models of establishment using effective area at PP_GRASS_. All top models that in sum include at least 90% AICc weights shown. EA= effective area. Climate variables abbreviated to indicate decade followed by variable type, e.g. 1P = first decade precipitation, 2T=second decade temperature, etc.

| **Intercept** | **EA** | **Log(EA)** | **1P** | **1T** | **2P** | **2T** | **3P** | **3T** | **Df** | **logLik** | **AICc** | **Delta** | **Weight** |
| --- | --- | --- | --- | --- | --- | --- | --- | --- | --- | --- | --- | --- | --- |
| **+** | **-** | **+** |  |  |  |  |  |  | 4 | -64.86744 | 137.734879 | 2.84093994 | 0.00998 |
| **+** | **-** | **+** | **+** |  |  |  |  |  | 5 | -64.093585 | 138.18717 | 3.29323109 | 0.00796 |
| **+** | **-** | **+** |  |  |  |  |  | **+** | 5 | -64.137513 | 138.275027 | 3.38108788 | 0.00761 |
| **+** | **-** | **+** |  |  | **+** |  |  |  | 5 | -64.569762 | 139.139525 | 4.24558581 | 0.00494 |
| **+** | **-** | **+** | **+** |  | **+** |  |  |  | 6 | -63.716897 | 139.433795 | 4.53985568 | 0.0042 |
| **+** | **-** | **+** | **+** |  |  |  |  | **+** | 6 | -63.742109 | 139.484218 | 4.59027843 | 0.00416 |
| **+** | **-** | **+** |  | **+** |  |  |  |  | 5 | -64.773279 | 139.546557 | 4.65261829 | 0.00402 |
| **+** | **-** | **+** |  |  |  | **+** |  |  | 5 | -64.863442 | 139.726885 | 4.83294543 | 0.00368 |
| **+** | **-** | **+** |  |  |  |  | **+** |  | 5 | -64.865245 | 139.73049 | 4.83655107 | 0.00367 |
| **+** | **-** | **+** |  |  | **+** |  |  | **+** | 6 | -63.930131 | 139.860263 | 4.96632366 | 0.00344 |
| **+** | **-** | **+** | **+** |  |  |  | **+** |  | 6 | -64.050043 | 140.100086 | 5.20614671 | 0.00307 |
| **+** | **-** | **+** | **+** | **+** |  |  |  |  | 6 | -64.073071 | 140.146141 | 5.25220217 | 0.00299 |
| **+** | **-** | **+** |  |  |  | **-** |  | **+** | 6 | -64.080216 | 140.160432 | 5.26649295 | 0.00298 |
| **+** | **-** | **+** | **+** |  |  | **-** |  |  | 6 | -64.093572 | 140.187144 | 5.29320512 | 0.00293 |
| **+** | **-** | **+** |  |  |  |  | **+** | **+** | 6 | -64.125648 | 140.251296 | 5.35735648 | 0.00283 |
| **+** | **-** | **+** |  | **+** |  |  |  | **+** | 6 | -64.12644 | 140.25288 | 5.35894103 | 0.00283 |
| **+** | **-** | **+** |  | **+** | **+** |  |  |  | 6 | -64.422893 | 140.845787 | 5.95184767 | 0.00210 |
| **+** | **-** | **+** | **+** |  | **+** |  |  | **+** | 7 | -63.459362 | 140.918723 | 6.02478403 | 0.00203 |
| **+** | **-** | **+** |  |  | **+** |  | **+** |  | 6 | -64.564546 | 141.129092 | 6.23515295 | 0.00183 |
| **+** | **-** | **+** |  |  | **+** | **+** |  |  | 6 | -64.566192 | 141.132383 | 6.23844413 | 0.00182 |
| **+** | **-** | **+** | **+** |  | **+** |  | **+** |  | 7 | -63.656824 | 141.313649 | 6.41970965 | 0.00167 |

**Species-wide mixed models using effective area.**

**Table S19.** Top effective area (EA) species-wide models of establishment for all *P. ponderosa* populations, with population (S) as a random effect. Climate variables abbreviated to indicate decade followed by variable type, e.g. 1p = first decade precipitation, 2T = second decade temperature, etc. Here we refer to population as site (S), so as not to confuse with precip (P) variables. * indicates that the term was included (if a random effect), and + / - indicates the sign (if a fixed effect).

| **Intercept** | **S** | **EA** | **Ln(EA)** | **1P** | **1T** | **2P** | **2T** | **3P** | **3T** | **EA*S** | **Ln(EA)*S** | **1P*S** | **1T*S** | **2P*S** | **2T*S** | **3P*S** | **3T*S** | **df** | **logLik** | **AICc** | **delta** | **weight** |
| --- | --- | --- | --- | --- | --- | --- | --- | --- | --- | --- | --- | --- | --- | --- | --- | --- | --- | --- | --- | --- | --- | --- |
| **+** | ***** | **-** | **+** |  |  |  | **-** |  |  | ***** |  |  |  |  |  |  |  | 11 | -325.70429 | 673.408576 | 0 | 0.013 |
| **+** | ***** | **-** | **+** |  |  |  | **-** |  | **-** | ***** |  |  |  |  |  |  | ***** | 15 | -321.74383 | 673.487651 | 0.0790746 | 0.013 |
| **+** | ***** | **-** | **+** |  |  |  | **-** |  |  |  | ***** |  |  |  |  |  |  | 11 | -325.89084 | 673.781672 | 0.3730957 | 0.011 |
| **+** | ***** | **-** | **+** |  |  |  | **-** |  | **-** | ***** |  |  |  |  |  |  |  | 12 | -324.92168 | 673.843368 | 0.4347919 | 0.011 |
| **+** | ***** | **-** | **+** |  |  |  | **-** |  | **-** |  | ***** |  |  |  |  |  | ***** | 15 | -321.97999 | 673.959971 | 0.5513951 | 0.010 |

**Table S20.** Top effective area (EA) species-wide models of establishment for all *P. flexilis* populations, with population (S) as a random effect. Climate variables abbreviated to indicate decade followed by variable type, e.g. 1p = first decade precipitation, 2T = second decade temperature, etc. Here we refer to population as site (S), so as not to confuse with precip (P) variables. * indicates that the term was included (if a random effect), and + / - indicates the sign (if a fixed effect).

| **Intercept** | **S** | **EA** | **Ln(EA)** | **1P** | **1T** | **2P** | **2T** | **3P** | **3T** | **EA*S** | **Ln(EA)*S** | **1P*S** | **1T*S** | **2P*S** | **2T*S** | **3P*S** | **3T*S** | **df** | **logLik** | **AICc** | **delta** | **weight** |
| --- | --- | --- | --- | --- | --- | --- | --- | --- | --- | --- | --- | --- | --- | --- | --- | --- | --- | --- | --- | --- | --- | --- |
| **+** | ***** | **+** | **+** |  | **-** |  |  |  | **-** | ***** | ***** |  | ***** |  |  |  | ***** | 11 | -139.59644 | 301.192872 | 0 | 0.013 |
| **+** | ***** | **+** | **+** |  | **-** |  |  |  | **-** |  | ***** |  | ***** |  |  |  | ***** | 10 | -140.59748 | 301.194964 | 0.00209 | 0.013 |
| **+** | ***** | **+** | **+** |  | **-** | **-** |  |  | **-** | ***** | ***** |  | ***** | ***** |  |  | ***** | 13 | -137.67359 | 301.347177 | 0.15430 | 0.012 |
| **-** | ***** | **+** | **+** |  | **-** |  | **-** |  | **-** |  | ***** |  | ***** |  | ***** |  | ***** | 12 | -138.70393 | 301.40787 | 0.21499 | 0.0121 |

**Table S21. Performance of population models. Mean absolute value of raw and proportional deviations from median stochastic simulations to observed numbers by model type and population, and proportion of observed values outside of 90% confidence interval predictions.**

| **population** | **model type** | **mean absolute deviation** | **mean absolute proportional deviation (actual / median prediction)** | **proportion of predictions outside 90% envelope (post-five trees)** |
| --- | --- | --- | --- | --- |
| PF ANCHOR | best | 68.62 | 0.821 | 0.000 |
| PF CASTLE | best | 4.155 | 1.264 | 0.023 |
| PP ANCHOR | best | 1.963 | 1.077 | 0.000 |
| PP CASTLE | best | 4.106 | 1.025 | 0.000 |
| PP COTTON | best | 32.25 | 1.072 | 0.211 |
| PP GRASS | best | 6.953 | 0.629 | 0.186 |
| PF ANCHOR | five trees | 70.056 | 1.608 | 0.667 |
| PF CASTLE | five trees | 8.107 | 0.847 | 0.214 |
| PP ANCHOR | five trees | 11.200 | 1.226 | 0.400 |
| PP CASTLE | five trees | 27.913 | 1.946 | 0.652 |
| PP COTTON | five trees | 36.061 | 1.046 | 0.273 |
| PP GRASS | five trees | 4.059 | 0.983 | 0.000 |
| PF ANCHOR | no climate | 25.360 | 0.660 | 0.280 |
| PF CASTLE | no climate | 3.750 | 1.109 | 0.000 |
| PP ANCHOR | no climate | 3.278 | 1.092 | 0.000 |
| PP CASTLE | no climate | 4.061 | 0.876 | 0.000 |
| PP COTTON | no climate | 70.197 | 1.876 | 0.211 |
| PP GRASS | no climate | 9.172 | 0.611 | 0.281 |
| PF ANCHOR | no density | 149.621 | 0.468 | 0.560 |
| PF CASTLE | no density | 14.357 | 2.011 | 0.762 |
| PP ANCHOR | no density | 18.870 | 2.073 | 0.741 |
| PP CASTLE | no density | 35.242 | 3.932 | 0.909 |
| PP COTTON | no density | 245.066 | 8.956 | 0.947 |
| PP GRASS | no density | 38.500 | 0.698 | 0.594 |
| PF ANCHOR | non-stochastic | 47.731 | 0.356 | NA |
| PF CASTLE | non-stochastic | 13.301 | 0.576 | NA |
| PP ANCHOR | non-stochastic | 15.510 | 0.351 | NA |
| PP CASTLE | non-stochastic | 16.059 | 0.421 | NA |
| PP COTTON | non-stochastic | 176.200 | 0.702 | NA |
| PP GRASS | non-stochastic | 12.006 | 0.368 | NA |

**Table S22. Performance of species-wide models (mixed models for each species separately, with site as a random effect). Mean absolute value of raw and proportional deviations from median stochastic simulations to observed numbers by model type and population, and proportion of observed values outside of 90% confidence interval predictions.**

| **population** | **model type** | **mean absolute deviation** | **mean absolute proportional deviation** | **proportion of predictions outside 90% envelope** |
| --- | --- | --- | --- | --- |
| PF ANCHOR | Best species-wide model | 10.54 | 1.348 | 0.00 |
| PF CASTLE | Best species-wide model | 3.333 | 1.089 | 0.00 |
| PP ANCHOR | Best species-wide model | 7.518 | 0.711 | 0.037 |
| PP CASTLE | Best species-wide model | 9.265 | 1.361 | 0.088 |
| PP COTTON | Best species-wide model | 31.039 | 1.074 | 0.211 |
| PP GRASS | Best species-wide model | 10.672 | 0.590 | 0.656 |
| PF ANCHOR | No climate | 47.48 | 0.541 | 0.520 |
| PF CASTLE | No climate | 3.821 | 1.047 | 0.000 |
| PP ANCHOR | No climate | 6.648 | 0.836 | 0.037 |
| PP CASTLE | No climate | 8.427 | 1.304 | 0.000 |
| PP COTTON | No climate | 47.000 | 1.598 | 0.231 |
| PP GRASS | No climate | 10.344 | 0.595 | 0.500 |
| PF ANCHOR | No density | 97.100 | 0.537 | 0.240 |
| PF CASTLE | No density | 42.393 | 1.220 | 0.000 |
| PP ANCHOR | No density | 28.241 | 5.240 | 0.481 |
| PP CASTLE | No density | 12.265 | 1.324 | 0.147 |
| PP COTTON | No density | 68.987 | 1.549 | 0.179 |
| PP GRASS | No density | 4.703 | 0.769 | 0.188 |
| PF ANCHOR | No site | 60.04 | 3.653 | 0.440 |
| PF CASTLE | No site | 43.714 | 0.973 | 0.500 |
| PP ANCHOR | No site | 6.981 | 1.008 | 0.556 |
| PP CASTLE | No site | 3.412 | 0.960 | 0.529 |
| PP COTTON | No site | 215.038 | 3.847 | 0.821 |
| PP GRASS | No site | 12.797 | 0.667 | 0.250 |
| PF ANCHOR | Non-stochastic | 30.559 | 2.504 | NA |
| PF CASTLE | Non-stochastic | 11.114 | 0.982 | NA |
| PP ANCHOR | Non-stochastic | 16.918 | 0.432 | NA |
| PP CASTLE | Non-stochastic | 22.343 | 0.563 | NA |
| PP COTTON | Non-stochastic | 87.690 | 0.528 | NA |
| PP GRASS | Non-stochastic | 12.212 | 0.463 | NA |

**Table S23. Top population models with and without density effects. Models were constructed using JAGS, and density effects constrained by forcing the coefficient of ln(mature trees) to be equal to one [constrained] or not [unconstrained]. A coefficient of one means that the # of recruits is a constant function of # mature trees through time (a coefficient < 1 or >1 indicates negative or positive density dependence, respectively). Unconstrained model rows shaded in blue. PF = Pinus flexilis, PP = pinus ponderosa. Median coefficient estimates are given followed by 5^th^ and 95^th^ percentile values in parentheses.**

| **Population** | **Constrained?** | **Intercept** | **Ln(MT)** | **First10T** | **First10P** | **First20T** | **First20P** | **First30T** | **First30P** | **r.newplts** | **deviance** | **DIC** |
| --- | --- | --- | --- | --- | --- | --- | --- | --- | --- | --- | --- | --- |
| **PF Castle** | Yes | -3.089 (-4.862, -1.301) | 1.000 (0.998, 1.001) |  |  |  | 9.586e-03 (-0.003, 2.235e-02) |  |  | 3.037e+01 (2.643, 9.172e+01) | 1.488e+02 | 151.806 |
| **PF Castle** | No | -3.466 (-5.325, -1.563) | 1.119 (0.865, 1.379) |  |  |  | 9.360e-03 (-0.003, 2.153e-02) |  |  | 3.716e+01 (3.027, 9.334e+01) | 1.488e+02 | 152.798 |
| **PP Castle** | Yes | -1.789 (-2.191, -1.329) | 9.9998e-01 (0.998, 1.002) |  |  | -9.609e-01 (-2.226, 4.998e-01) |  |  |  | 3.144 (0.704, 2.899e+01) | 1.540e+02 | 161.292 |
| **PP Castle** | No | -3.598e-01 (-0.934, 2.252e-01) | 5.272e-01 (0.372, 6.890e-01) |  |  | -1.122 (-1.868, -3.626e-01) |  |  |  | 5.338e+01 (10.967, 9.999e+01) | 1.336e+02 | 137.552 |
| **PP Anchor** | Yes | -8.872e-01 (-3.903, 2.047) | 9.999e-01 (9.981e-01, 1.002) | 1.315 (4.176e-01, 2.262) | 6.864 (-2.016e+03, 1.874e+03) |  | -1.255e-02 (-2.639e-02, 6.968e-04) | -1.306 (-2.072, -5.427e-01) | 9.796e-03(-6.182e-03, 2.613e-02) | 6.288e+01 (1.551e+01, 9.998e+01) | 9.952e+01 | 105.539 |
| **PP Anchor** | No | -1.045 (-4.299, 1.877) | 7.702e-01 (5.758e-01, 9.724e-01) | 1.511 (5.609e-01, 2.451) | -1.241e+01 (-1.935e+03, 1.946e+03) |  | -9.899e-03 (-2.323e-02, 3.451e-03) | -1.090 (-1.862, -2.702e-01) | 1.401e-02 (-2.105e-03, 3.101e-02) | 5.821e+01 (1.538e+01, 9.976e+01) | 9.553e+01 | 102.458 |
| **PF Anchor** | Yes | -2.436 (-7.121, 2.145) | 1.000 (0.998, 1.002) | -1.088 (-2.828, 7.073e-01) | 1.537e-02 (-0.018, 4.980e-02 ) |  |  | 1.951 (-3.987, -1.640e-02) |  | 9.388e-01 (0.236, 2.282) | 1.357e+02 | 143.133 |
| **PP Anchor** | No | -6.172 (-12.034, -1.149) | 1.977 (1.424, 2.663) | -2.263 (-4.108, -5.364e-01) | 2.354e-02 (-0.009, 5.933e-02) |  |  | -4.679 (-7.516, -2.083) |  | 1.645 (0.452, 3.624) | 1.215e+02 | 132.405 |
| **PP Grass** | Yes | -5.445e-01 (-1.242, 2.071e-01) | 1.000 (0.998, 1.001) |  |  |  |  |  |  | 3.778e-01 (0.190, 6.205e-01) | 1.538e+02 | 156.704 |
| **PP Grass** | No | 5.023e-01 (-2.2038e-02, 1.033) | 1.200e-01 (-8.104e-02, 3.178e-01) |  |  |  |  |  |  | 3.070 (3.136e-01, 6.753e+01) | 1.271e+02 | 132.277 |
| **PP Cotton** | Yes | -1.330 (-1.627, -1.004) | 1.000 (0.998, 1.002) |  |  |  |  |  |  | 1.611 (0.942, 2.477) | 2.992e+02 | 301.91 |
| **PP Cotton** | No | 5.128e-01 (-8.765e-02, 1.114) | 5.445e-01 (4.181e-01, 6.792e-01) |  |  |  |  |  |  | 3.650 (1.847, 5.950) | 2.695e+02 | 273.47 |
|  |  |  |  |  |  |  |  |  |  |  |  |  |

**Table S24. Top species-wide models with and without density effects. Models were constructed using JAGS, and density effects constrained by forcing the coefficient of ln(mature trees) to be equal to 1 [constrained] or not [unconstrained]. A coefficient of 1 means that the # of recruits is a constant function of # mature trees through time (a coefficient < 1 or >1 indicates negative or positive density dependence, respectively). Unconstrained model rows shaded in blue. PF = Pinus flexilis, PP = pinus ponderosa. Median coefficient estimates are given followed by 5^th^ and 95^th^ percentile values in parentheses. For fixed Site effects, the range of median values is provided for PP populations (minimum – maximum), and both values are provided for PF populations (value for PF anchor; value for PP anchor).**

| **Spp** | **Constrained?** | **Intercept** | **Ln(MT)** | **Site** | **Site * Ln(MT)** | **First10T** | **First10P** | **First20P** | **First30T** | **First30P** | **Site * first10P** | **Site * first10T** | **Site * first30P** | **Site * first 30 T** | **r.newplts** | **deviance** | **DIC** |
| --- | --- | --- | --- | --- | --- | --- | --- | --- | --- | --- | --- | --- | --- | --- | --- | --- | --- |
| **PP** | Yes | -1.340  (-2.566, -7.864e-02) | 1.000 (9.980e-01,  1.001) | 7.242e-06 −  1.598 | -8.785e-01 −  -2.599e-05 |  | 1.750e-03  (-7.117e-03,  1.065e-02) |  |  |  |  |  |  |  | 3.768 (2.229, 5.563) | 654.3 | 664.768 |
| **PP** | No | -5.933e-01  (-1.973, 8.313e-01) | 7.191e-01 (4.767e-01, 9.713e-01) | -6.652e-03 −  8.556e-01 | -8.328e-01 −  3.268e-06 |  | 1.729e-03  (-7.113e-03,  1.082e-02) |  |  |  |  |  |  |  | 3.917 (2.393, 5.853) | 652.1 | 663.640 |
| **PF** | Yes | -1.211  (-5.755, 2.597) | 1.000 (9.980e-01, 1.002) | -1.578e-05 ; -1.345 | 2.120e-05;  -1.535e-02 | 7.062 e-01 (-2.646,  -1.653e-01) | 1.540e-03  (-1.148e-02,  2.928-02) | 3.772e-03  (-1.341e-02, 1.996e-02) | 1.248  (-2.812,  -4.307e-02) | 3.440e-05  (-1.496e-02,  1.649e-02) | 2.596e-05;  -8.285e-01 | -7.674e-06;  -9.548e-01 | -5.315e-06; 1.912e-01 | 7.037e-06; -1.294 | 1.751 (7.529e-01,  3.343) | 300.1 | 315.485 |
| **PF** | No | -3.516  (-8.906, 2.090e-01) | 1.730 (1.261, 2.233) | -1.736e-05;  1.317 | -1.152e-06;  -4.292e-01 | -1.645  (-3.193, 5.345e-02) | 2.342e-03  (-5.190e-03,  3.542e-02) | 3.876e-03  (-1.200e-02, 2.005e-02) | -3.673  (-5.676,  -1.821) | 3.514e-05 (-1.713e-02,  -1.189e-02) | -2.303e-06;  -1.158 | 6.491e-06; -1.039 | 7.855e-06; -3.236 | 3.248e-06; -1.058 | 2.383 (1.012, 4.436) | 281.9 | 299.426 |

**Table S25. Correlation matrix of climate variables.**

|  | **10yr precipitation** | **10 year temperature** | **20 year precipitation** | **20 year temperature** | **30 year precipitation** | **30 year temperature** |
| --- | --- | --- | --- | --- | --- | --- |
| **10yr precipitation** | 1 | -0.008 | -0.031 | 0.024 | -0.111 | 0.302 |
| **10yr temperature** |  | 1 | -0.059 | 0.459 | -0.230 | 0.286 |
| **20yr precipitation** |  |  | 1 | -0.017 | -0.041 | 0.0315 |
| **20yr temperature** |  |  |  | 1 | -0.019 | 0.421 |
| **30yr precipitation** |  |  |  |  | 1 | 0.018 |
| **30yr temperature** |  |  |  |  |  | 1 |

**References**

Baddeley, A., Turner, R. (2005). Spatstat: An R package for Analyzing Spatial Point Patterns. *Journal of Statistical Software,* *12*(6), 1-42.

Baddeley, A., Diggle, P.J., Hardegen, A., Lawrence, T., Milne, RK., Nair, G. (2014). On tests of spatial pattern based on simulation envelopes. *Ecological Monographs*, *84*(3), 477-489.

Committee on the Status of Endangered Wildlife in Canada (COSEWIC). (2014). COSEWIC Annual Report.

Diggle, P.J. (2014). Statistical Analysis of Spatial and Spatio-Temporal Point Patterns (3^rd^ Edition). Boca Raton, Florida: Chapman & Hall / CRC monographs on Statistics and Applied Probability.

Rigby, R.A., Stasinopoulos, D.M. 2005. “Generalized additive models for location, scale, and shape, (with discussion).” *Applied Statistics* **54**(3): 507-554.

Oliver, W.W., Ryker, R.A. (1990). *Pinus ponderosa* Dougl. Ex Laws**.** *In* Burns, Russell M.; Honkala, Barbara H., technical coordinators. Silvics of North America, Volume 1. Conifers. Agriculture Handbook 654. Washington D.C.: U.S. Department of Agriculture. pp 413-424.

Wiegand, T., Moloney, K.A. (2004). Rings, circles, and null-models for point pattern analysis in ecology. *Oikos, 104*(2), 209-229.
